# Supplementary material for: Deubiquitination of BES1 by UBP12/UBP13 promotes brassinosteroid signaling and plant growth
Source: Plant Commun. 2022 Jun 15;3(5):100348. doi: 10.1016/j.xplc.2022.100348 (PMC9483116; doi:10.1016/j.xplc.2022.100348)
Supplement: Document S1. Supplemental Figures 1–11 and Supplemental Tables 1 and 2 [file mmc1.pdf]

Plant Communications, Volume *3*

Supplemental information

Deubiquitination of BES1 by UBP12/UBP13 promotes brassinosteroid signaling and plant growth

Su-Hyun Park, Jin Seo Jeong, Yu Zhou, Nur Fatimah Binte Mustafa, and Nam-Hai Chua

Supplemental information

Deubiquitination of BES1 by UBP12/UBP13 promotes brassinosteroid signaling and plant growth

Su-Hyun Park[1], Jin Seo Jeong[1,2], Yu Zhou[1], Nur Fatimah Binte Mustafa[1], Nam-Hai Chua[1,*]

[1]Temasek Life Sciences Laboratory, 1 Research Link, National University of Singapore, Singapore 117604, Singapore

[2] Present address: Wilmar International Limited, 28 Biopolis Road, Singapore 138568, Singapore

*Corresponding author: Nam-Hai Chua; chua@rockefeller.edu

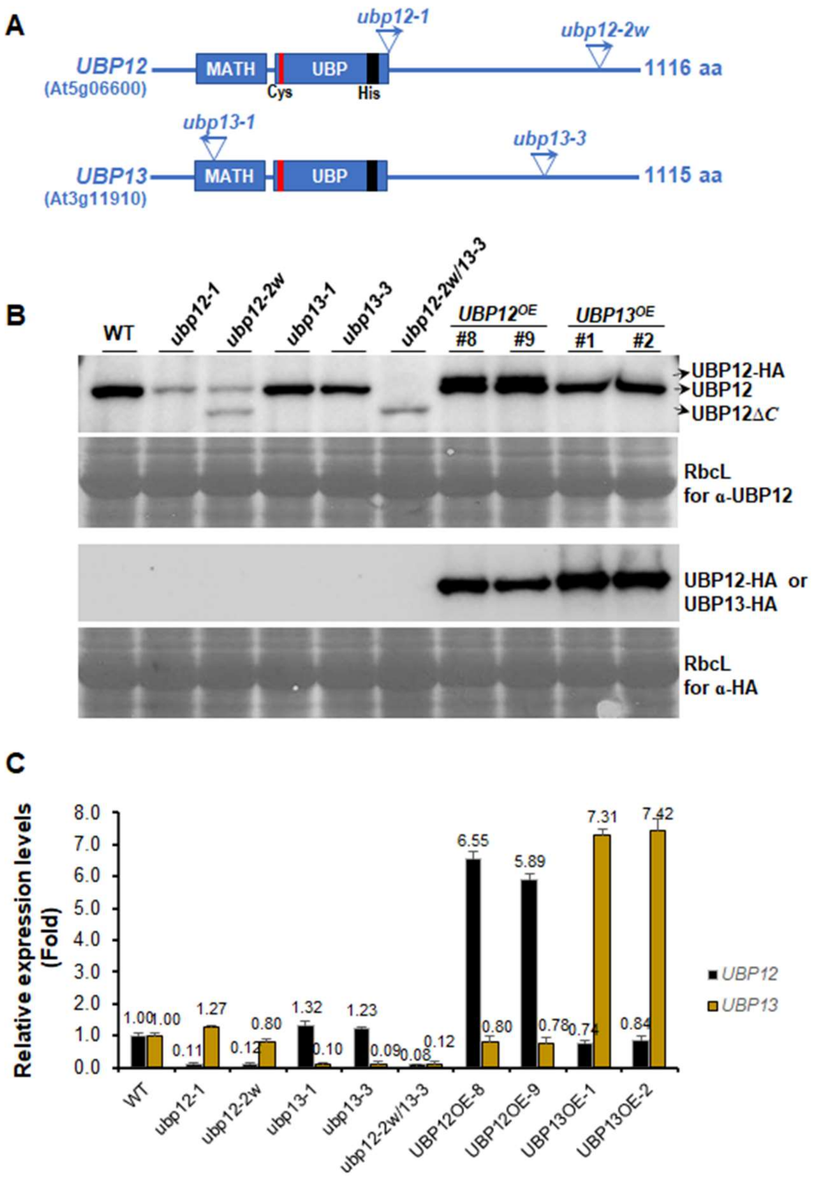

Figure S1. Molecular characterization of plants deficient in UBP12/UBP13 and plants overexpressing these proteins.

(A) Schematic gene structures of *UBP12* and *UBP13* with T-DNA insertions and encoded protein domains. MATH, meprin and tumor necrosis factor receptor-associated factor homology domain; UBP, Ubiquitin-specific protease domain containing Cys and His motifs. **(B)** UBP12/UBP13 protein levels in 4 single mutants, *ubp12-1*, *ubp12-2w*, *ubp13-1*, *ubp13-3*, one double mutant, *ubp12-2w/13-3,* and 2 independent transgenic lines over-expressing *UBP12* (*UBP12^{OE}*) or *UBP13* (*UBP13^{OE}*). Seven-day-old seedlings grown under normal conditions were used. Endogenous UBP12 and UBP13 were detected by immunoblots using anti-UBP12

antibody, which reacted with both homologous proteins. Transgenic UBP12-HA and UBP13-HA in *UBP12^{OE}* and *UBP13^{OE}* plants were detected using an anti-HA antibody. UBP12Δ*C* indicates a truncated form of UBP12 due to the T-DNA insertion in *ubp12-2w*. RbcL protein levels were used as a loading control. Western blot assays were analyzed in three independent experiments and images from one representative set were shown. **(C)** Relative gene expression levels of *UBP12* and *UBP13* in various plants. Transcript levels of *UBP12* and *UBP13* were analyzed in 7-d-old seedlings by qRT-PCR analysis. Expression levels of WT were set as 1. All qRT-PCR experiments were performed in triplicates. Average values of 3 independent biological samples (*n*=3) were presented with standard deviation. *ACT2* expression levels were used as a control for normalization.

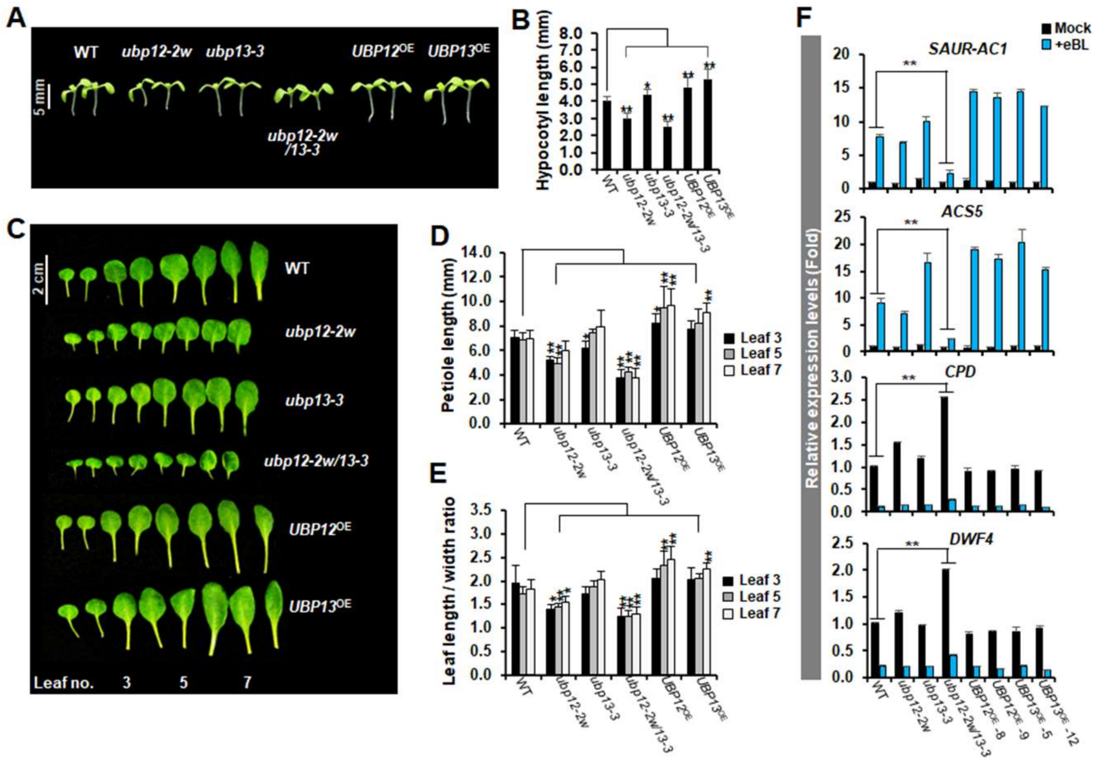

Figure S2. BR-deficient phenotypes of *ubp12-2w/13-3* plants.

(A) Phenotypes of WT (Col-0), *ubp12-2w*, *ubp13-3*, *ubp12-2w/13-3,* and *UBP12*- or *UBP13*-overexpressing plants (*UBP12^{OE}* or *UBP13^{OE}*). Seedlings were grown on ½ MS medium under dim light (25 µmol $m^{-2}s^{-1}$) for 7 days. Bar, 5 mm. **(B)** Hypocotyl lengths of seedlings indicated in **(A)**. **(C-E)** Phenotypes of rosette leaves of various genotypes grown on ½ MS medium for 3 weeks under normal light (100 µmol $m^{-2}s^{-1}$). Bar, 2 cm. The petiole length (mm) and the ratio of leaf length to width of the 3rd, 5th, and 7th leaves are shown in **(D)** and **(E)**. Fifteen independent biological samples (*n*=15) were analyzed and one image is shown as a representative. *, $P < 0.05$; **, $P < 0.01$ (two-tailed *t-test*). **(F)** Changes in BR-responsive gene expression in plants deficient in or overexpressing UBP12/UBP13. Transcript levels of genes repressed- (*CPD* and *DWF4*) or induced- (*SAUR-AC1* and *ACS5*) by brassinosteroids (BR) were analyzed in 7-d-old seedlings treated with 1 µM of epi-brassinolide (eBL) for 6 hours by qRT-PCR analysis. Expression levels of WT were set as 1. All qRT-PCR experiments were performed in triplicates. Average values of 3 independent biological samples (*n*=3) were presented with standard deviation. *ACT2* expression levels were used as a control for normalization.

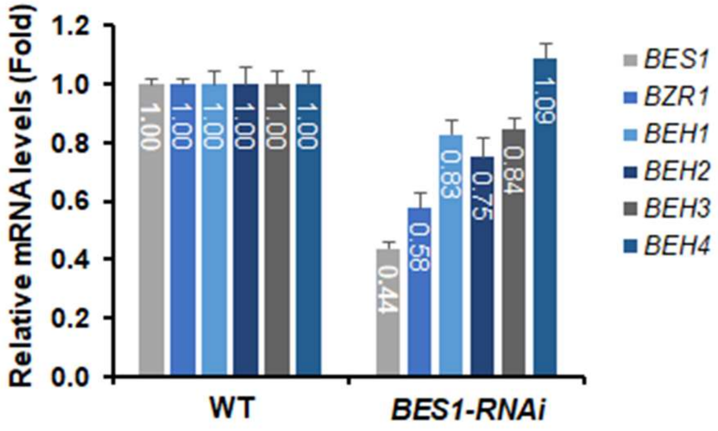

Figure S3. Expression of *BES1* family genes in *BES1-RNAi* lines.

Transcript levels of *BES1* family genes were analysed by qRT-PCR using 7-day-old seedlings grown in normal conditions. All qRT-PCR experiments were performed in triplicates and the average values were presented with S.D. (standard deviation) (n=3). *ACT2* expression levels were used as a control for normalization.

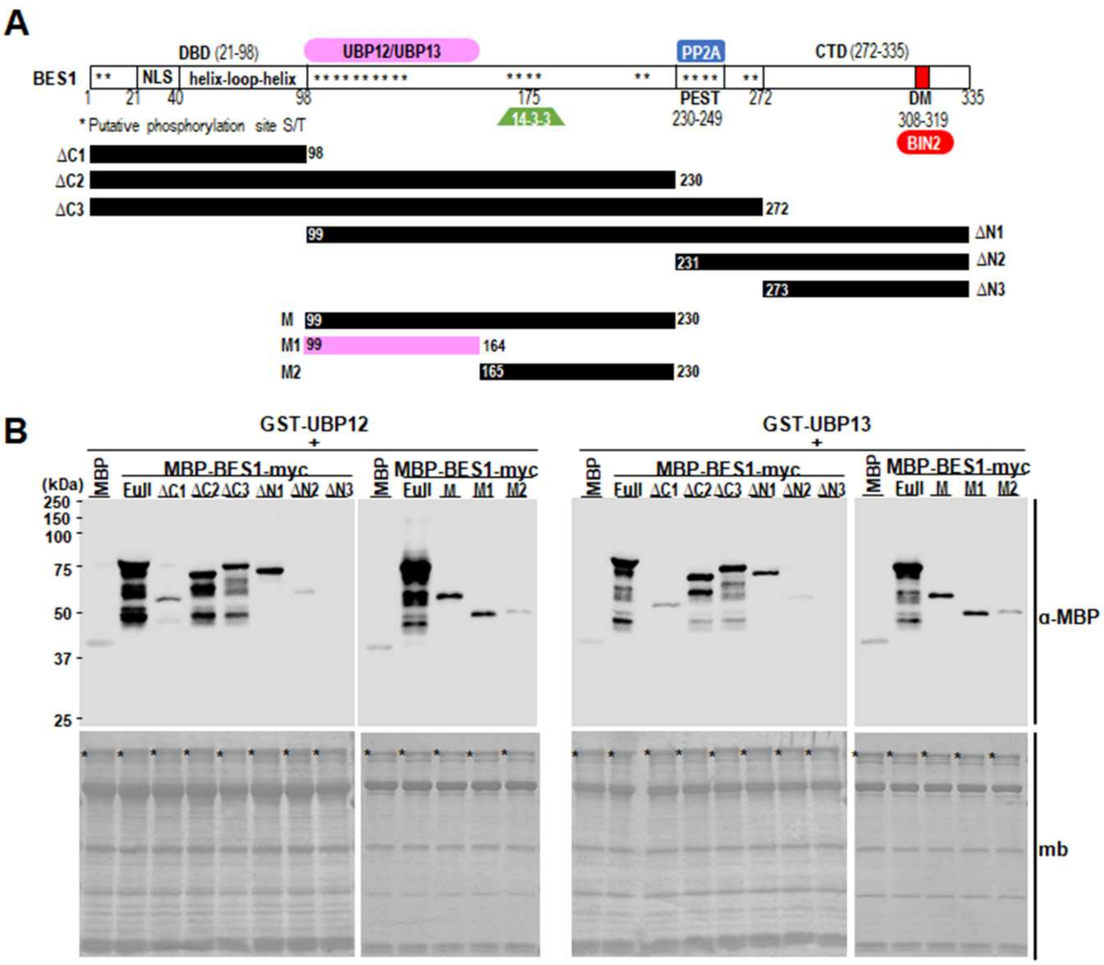

Figure S4. Interaction of UBP12/UBP13 with BES1 fragments.

(A) Schematic diagram of various truncated forms of BES1 (upper panel). Numbers indicate the amino acid positions of the derivatives. **(B)** Truncated forms of BES1 like ΔC1, ΔC2, ΔC3, ΔN1, ΔN2, and ΔN3, and the middle part of BES1 (M) were dissected into two regions like M1 and M2, which were pulled down by GST-UBP12 and GST-UBP13. 'Full' denotes the full-length BES1. MBP was used as a negative control. Two µg of each protein was used in all experiments. Protein bands were detected by immunoblots using anti-MBP antibody and the membrane (mb) was stained with Coomassie Brilliant Blue. The asterisk marks resin-bound bait proteins like GST-UBP12 and GST-UBP13. Three independent experiments were performed and images from one representative set are shown.

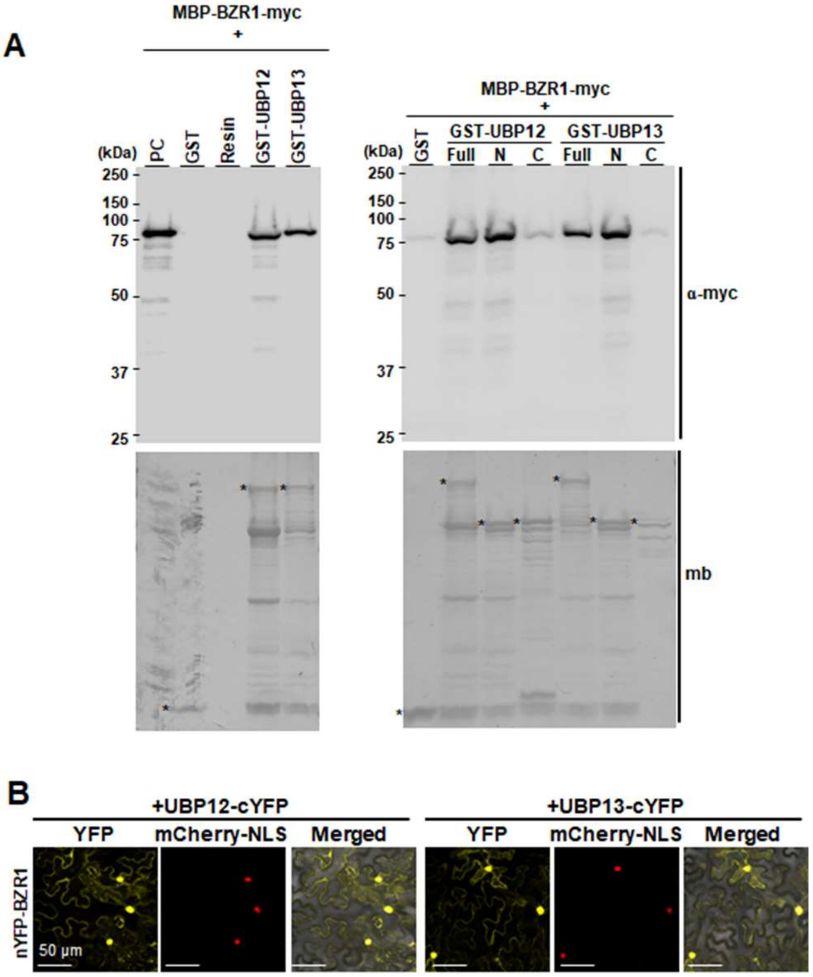

Figure S5. UBP12/UBP13 and BZR1 interaction *in vitro*.

(A) MBP-BZR1-myc was pulled down by GST resin-bound full-length UBP12/UBP13 (Full) and derivatives carrying the N- or C-terminal region (N or C) as shown in Figure 3A. Bands were detected by immunoblots using anti-myc antibody. Membrane (mb) was stained with Coomassie Brilliant Blue. Two µg of each protein was used in all experiments. The positive control (PC) sample contained 50 ng MBP-BZR1-myc. The asterisk marks resin-bound bait proteins. MBP, maltose-binding protein; GST, glutathione-S-transferase, kDa, kilodalton. **(B)** Interaction between UBP12/UBP13 and BZR1 in tobacco (*Nicotiana benthamiana*) leaves assayed by Bimolecular Fluorescence Complementation (BiFC). nYFP-BZR1 was transiently co-expressed with UBP12-cYFP or UBP13-cYFP in leaves of *N. benthamiana* infiltrated with *Agrobacterium*. Localization is shown by merging YFP, mCherry-NLS (Nuclear Localization Sequence) and DIC images (Merged). Confocal microscopic images were taken 3 days after infiltration. DIC, differential interference contrast, Bars, 50 µm. Western blots and BiFC assays were analyzed in 3 independent experiments and images of a representative set are shown.

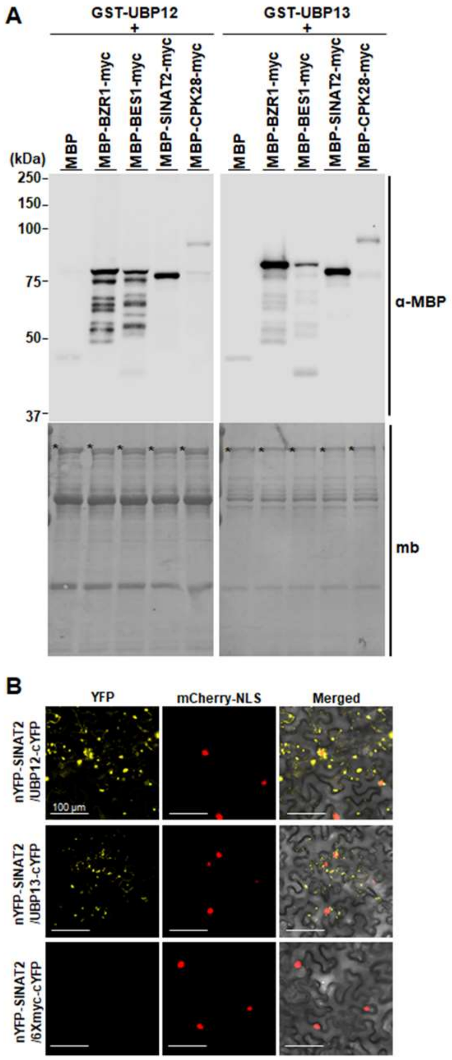

Figure S6. In vitro and in vivo interaction between UBP12/UBP13 and SINAT2.

(A) UBP12/UBP13 and SINAT2 interaction *in vitro*. MBP-SINAT2-myc was pulled down by UBP12/UBP13 tagged with GST. Protein bands were detected by immunoblots using anti-myc antibody. Membrane (mb) was stained with Coomassie Brilliant Blue. MBP and MBP-CPK28-myc were used as negative controls. MBP-BZR1-myc and MBP-BES1-myc were used as positive controls. Two µg of each protein was used in all experiments. The asterisk marks resin-bound bait proteins. MBP, maltose-binding protein; GST, glutathione-S-transferase, kDa, kilodalton. **(B)** In vivo interaction between UBP12/UBP13 and SINAT2 assayed by Bimolecular Fluorescence Complementation (BiFC). *SINAT2* fused with *nYFP* was transiently co-expressed with *UBP12-cYFP* and/or *UBP13-cYFP* in tobacco leaves (*N. benthamiana*) infiltrated with *Agrobacterium*. Localization is shown by merging YFP, mCherry-NLS (Nuclear Localization Sequence), and DIC images (Merged). Confocal microscopic images were taken 3 days after infiltration. DIC, differential interference contrast. Bars, 100 µm.

Western blots and BiFC assays were analyzed in 3 independent experiments and images of a representative set are shown.

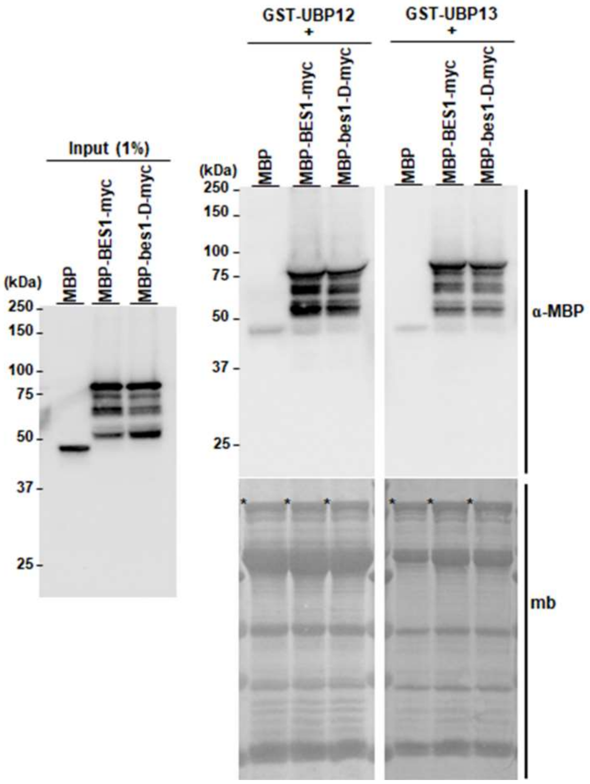

Figure S7. In vitro interaction between UBP12/UBP13 and bes1-D.

(A) UBP12/UBP13 and bes1-D interaction *in vitro*. MBP-bes1-D-myc was pulled down by UBP12/UBP13 tagged with GST. Protein bands were detected by immunoblots using anti-myc antibody. Membrane (mb) for GST-UBP12 and GST-UBP13 was stained with Coomassie Brilliant Blue. MBP was used as a negative control and MBP-BES1-myc was used as a positive control. Two µg of each protein was used in all experiments. The asterisk marks resin-bound bait proteins. MBP, maltose-binding protein; GST, glutathione-S-transferase, kDa, kilodalton.

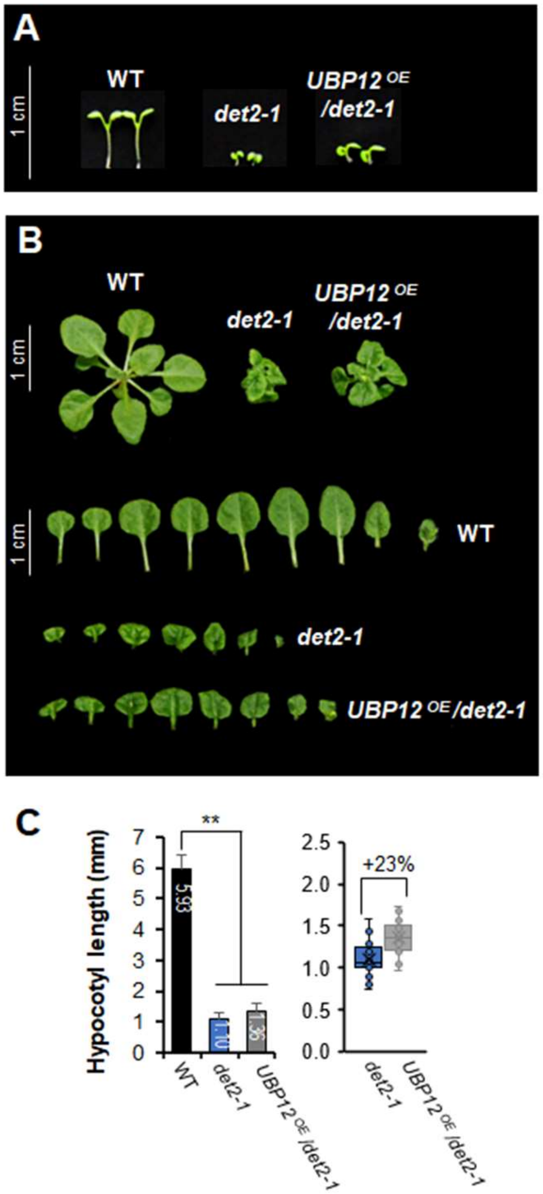

Figure S8. Partial restoration of *det2-1* BR-deficient phenotypes by *UBP12* overexpression.

(A) Seedlings for WT, *det2-1*, and *UBP12^{OE}/det2-1* were grown on ½ MS medium under dim light (25 µmol m^{-2}s^{-1}) for 7 days. The *UBQ10:UBP12-HA* construct was used for over-expression. Bar, 1 cm. **(B)** Phenotypes of rosette leaves of various genotypes grown on ½ MS medium for 3 weeks under normal light (100 µmol m^{-2}s^{-1}). Bar, 1 cm. **(C)** Hypocotyl lengths of seedlings indicated in **(A)**. Fifteen independent biological samples (n=15) were analyzed and one image is shown as a representative. *, $P < 0.05$; **, $P < 0.01$ (two-tailed *t-test*).

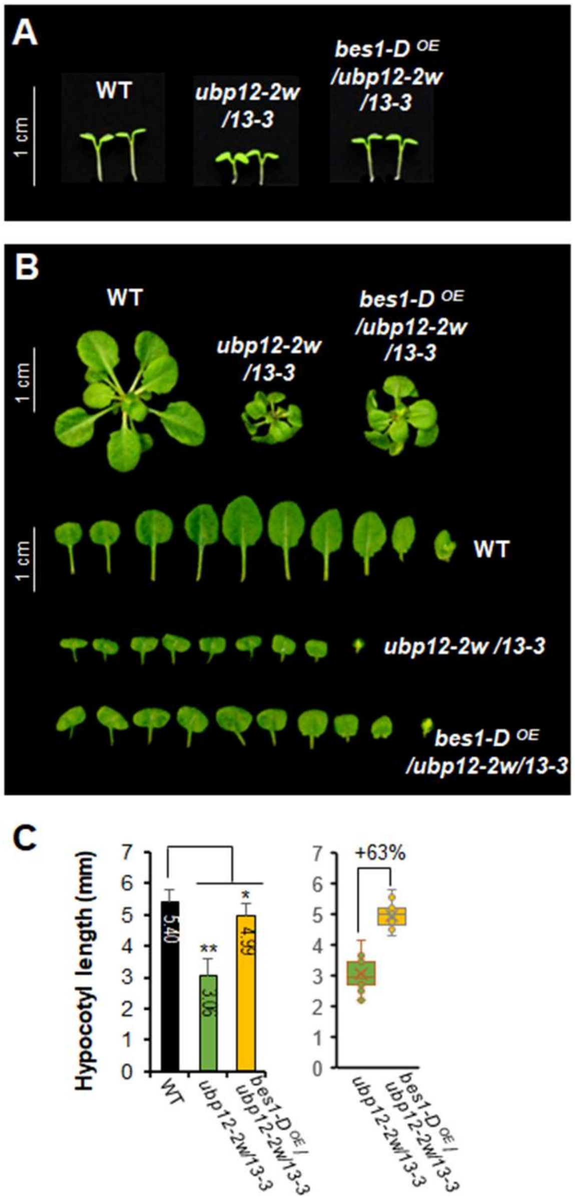

Figure S9. Partial restoration of *ubp12-2w/13-3* BR-deficient phenotypes by *bes1-D* overexpression.

(A) Seedlings for WT, *ubp12-2w/13-3* and *bes1-D*^{OE}*/ubp12-2w/13-3* were grown on ½ MS medium under dim light (25 µmol m^{-2}s^{-1}) for 7 days. *35S:bes1-D-YFP* construct was used for over-expression. Bar, 1 cm. **(B)** Phenotypes of rosette leaves of various genotypes grown on ½ MS medium for 3 weeks under normal light (100 µmol m^{-2}s^{-1}). Bar, 1 cm. **(C)** Hypocotyl lengths of seedlings indicated in **(A)**. Fifteen independent biological samples (n=15) were analyzed and one image is shown as a representative. *, $P < 0.05$; **, $P < 0.01$ (two-tailed *t-test*).

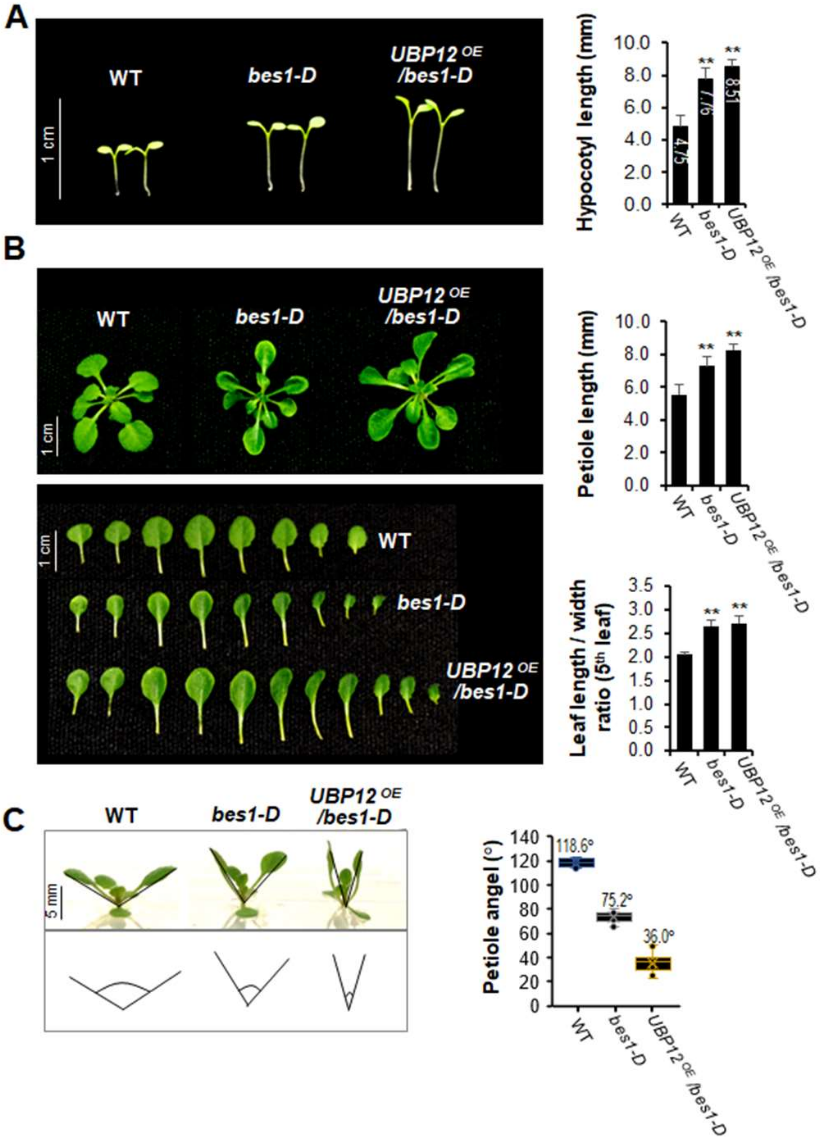

Figure S10. *UBP12* overexpression promotes growth of *bes1-D* plants.

"(A) Seedlings of WT, *bes1-D* (CS65988) and *UBP12*^{OE}/*bes1-D* were grown on ½ MS medium under dim light (25 µmol m^{-2}s^{-1}) for 7 days. The *UBQ10:UBP12-HA* construct was used for over-expression. Bar, 1 cm. Hypocotyl lengths (mm) of seedlings are indicated on the right panel. **(B)** Phenotypes of rosette leaves of various genotypes grown on ½ MS medium for 3 weeks under normal light (100 µmol m^{-2}s^{-1}). Bar, 1 cm. The petiole length (mm) and the ratio of leaf length to width of the 5th leaves are shown on the right panels. **(C)** Petiole angles of 2-week-old plants. Fifteen independent biological samples (n=15) were analyzed and one representative image is shown. *, $P < 0.05$; **, $P < 0.01$ (two-tailed *t-test*).

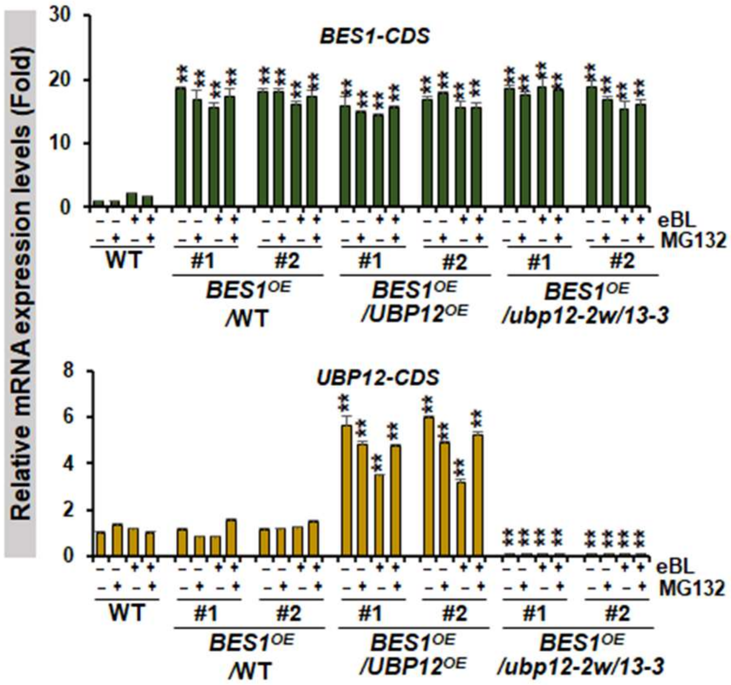

Figure S11. *BES1* and *UBP12* transcript levels of *BES1^{OE}*/WT, *BES1^{OE}/UBP12^{OE}*, and *BES1^{OE}/ubp12-2w/13-3*.

Quantitative RT-PCR analysis of *BES1* and *UBP12* transcript levels of *BES1^{OE}*/WT, *BES1^{OE}/UBP12^{OE}*, and *BES1^{OE}/ubp12-2w/13-3* treated with/without MG132 or eBL as shown in figure 5F. Samples prepared from whole seedlings were used for immunoblotting and qRT-PCR analysis. Transcript levels of *BES1* and *UBP12* were examined by qRT-PCR with primers specific for each coding sequence (CDS). All qRT-PCR experiments were performed in triplicates. Expression values of WT were set as 1. Average values from 3 independent biological experiments (n=3) were shown with standard deviation, SD (±). *ACT2* expression levels were used as a control for normalization. *, $P < 0.05$; **, $P < 0.01$ (two-tailed *t-test*).

Table S1 Primer list used in this study

Gene	Purpose	Primer Sequence	
		Forward	Reverse
UBP12	CDS	5'- ATGACTATGATGACTCCGCCTCCCGTTGAT -3'	5'- ATTGTATATTTTTACCGGCTTCTCGTAAGC -3'
UBP13	CDS	5'- ATGACTATGATGACTCCGCCGCCGCTAGAT -3'	5'- ATTGTATATTTTCACCGGCTTCTCGTATGC -3'
BES1	CDS	5'- ATGACGTCTGACGGAGCAA -3'	5'- ACTATGAGCTTTACCATTTCCAAGCG -3'
BZR1	CDS	5'- ATGACTTCGGATGGAGCTACG -3'	5'- ACCACGAGCCTTCCCATTTC -3'
SINAT2	CDS	5'- ATGGCTCCTGGAGGCAGT -3'	5'- CTCCTCTTTCCAGATTCGTCCAGT -3'
UBP12-N	derivatives	5'- ATGACTATGATGACTCCGCCTCCCGTTGAT -3'	5'- TAAATGTTCTGCTATGTCTTTCTCATC -3'
UBP12-C	derivatives	5'- CACCAGGGTGAGGCTAAAGAAAGAG -3'	5'- ATTGTATATTTTTACCGGCTTCTCGTAAGC -3'
UBP13-N	derivatives	5'- ATGACTATGATGACTCCGCCGCCGCTAGAT -3'	5'- CAAATGTTCCGCAATGTCTTTC -3'
UBP13-C	derivatives	5'- CACCCGGGTGAGGCTGAAAAAAG -3'	5'- ATTGTATATTTTCACCGGCTTCTCGTATGC -3'
BES1ΔC1	derivatives	5'- ATGACGTCTGACGGAGCAA -3'	5'- AGCCATGTCACCAGGTAGAG -3'
BES1ΔC2	derivatives	5'- ATGACGTCTGACGGAGCAA -3'	5'- ATGGAACTGGCGATGATGAG -3'
BES1ΔC3	derivatives	5'- ATGACGTCTGACGGAGCAA -3'	5'- CGAGGTTGGCACCATAGAGG -3'
BES1ΔN1	derivatives	5'- ATGGGATCATCTTCTCGAGCAACTCC -3'	5'- ACTATGAGCTTTACCATTTCCAAGCG -3'
BES1ΔN2	derivatives	5'- ATGGCTCCGGCTACTATACCTGAAT -3'	5'- ACTATGAGCTTTACCATTTCCAAGCG -3'
BES1ΔN3	derivatives	5'- ATGCCTACCTTCAATCTCGTGAAACCT -3'	5'- ACTATGAGCTTTACCATTTCCAAGCG -3'
BES1_M	derivatives	5'- ATGGGATCATCTTCTCGAGCAA -3'	5'- ATGGAACTGGCGATGATGAG -3'
BES1_M1	derivatives	5'- ATGGGATCATCTTCTCGAGCAA -3'	5'- AGGAAGCGATGAAGGAATACCA -3'
BES1_M2	derivatives	5'- ATGCCACTTAGAATCTCAAACAG -3'	5'- ATGGAACTGGCGATGATGAG -3'
CPD	qRT-PCR	5'- AGACGTGCAATGACGGATGT -3'	5'- TACCGAGTTGCTCTGCCATC -3'
DWF4	qRT-PCR	5'- CCCTAGTGGGTGGAAAGTGT -3'	5'- TCCGTTGTTTTGCTGTTGCC -3'
SAUR-AC1	qRT-PCR	5'- AGGGAATCATCGTCGACACC -3'	5'- AAGTATGAAACCGGCACCACA -3'
ACS5	qRT-PCR	5'- GGACTTCCTGGTTTCCGTGT -3'	5'- GTCGGAGAGCAATGCAGAGA -3'
BES1	qRT-PCR	5'- GATGACGTCTGACGGAGCAA -3'	5'- ACCAGTATAAATCTTCGCCGCA -3'
UBP12	qRT-PCR	5'- TTGCCAAGTGGAAGTTTGCG -3'	5'- CAACCCGAGGTACTGCTCAA -3'
UBP13	qRT-PCR	5'- GGGCAAAGCGTCAAAACCAT -3'	5'- CGGTCCACGCTCTATTTCCA -3'
ACT2	qRT-PCR	5'- AGTGGTCGTACAACCGGTATTGT -3'	5'- GATGGCATGAGGAAGAGAGAAAC -3'

Table S2 Phenotype changes of various genotypes

Genotypes	Hypocotyl length					Petiole length					Leaf length/width ratio				
	Average	ST	*P*-val	Percent vs WT (%)	Change	Average	ST	*P*-val	Percent vs WT (%)	Change	Average	ST	*P*-val	Percent vs WT (%)	Change
WT	4.9	0.5	1.00	100	0	8.9	0.3	1.00	100	0	2.1	0.1	1.00	100	0
BES1-RNAi	4.1	0.1	0.01	84	-16 ↓	4.8	0.3	0.00	54	-46 ↓	1.2	0.1	0.00	55	-45 ↓
ubp12-2w/13-3	3.2	0.5	0.00	64	-36 ↓	4.8	0.2	0.00	54	-46 ↓	1.4	0.1	0.00	63	-37 ↓
BES1-RNAi x ubp12-2w/13-3	3.3	0.3	0.04	66	-34 ↓	4.6	0.2	0.00	52	-48 ↓	1.3	0.0	0.00	59	-41 ↓
BES1^{OE} *#16*	5.7	0.4	0.01	116	16 ↑	9.9	0.5	0.06	111	11 ↑	2.8	0.1	0.03	131	31 ↑
BES1^{OE} *#18*	6.0	0.5	0.02	123	23 ↑	10.3	0.3	0.01	115	15 ↑	2.9	0.1	0.01	134	34 ↑
UBP12^{OE} *#8*	5.7	0.2	0.12	116	16 ↑	10.5	0.4	0.01	118	18 ↑	2.6	0.2	0.03	123	23 ↑
UBP12^{OE} *#9*	5.5	0.4	0.00	111	11 ↑	10.3	0.2	0.00	115	15 ↑	2.5	0.1	0.03	119	19 ↑
BES1^{OE}*/UBP12*^{OE} *#16*	7.1	0.8	0.00	145	**45** ↑	12.5	0.3	0.00	140	**40** ↑	3.1	0.3	0.01	145	**45** ↑
BES1^{OE}*/UBP12*^{OE} *#23*	7.2	0.6	0.01	146	**46** ↑	12.7	0.5	0.00	143	**43** ↑	3.2	0.2	0.00	151	**51** ↑
BES1^{OE}*/ubp12-2w/13-3 #6*	3.1	0.2	0.19	64	-36 ↓	4.7	0.4	0.00	53	-47 ↓	1.5	0.1	0.05	72	-28 ↓
BES1^{OE}*/ubp12-2w/13-3 #17*	3.5	0.3	0.49	71	-29 ↓	4.9	0.4	0.00	55	-45 ↓	1.6	0.1	0.06	74	-26 ↓
UBP12^{OE}*/RNAi-BES1 #3*	5.0	0.3	0.89	101	1	6.4	0.5	0.00	72	-28 ↓	1.7	0.1	0.01	79	-21 ↓
UBP12^{OE}*/RNAi-BES1 #9*	5.1	0.4	0.45	104	4	6.9	0.4	0.00	77	-23 ↓	1.8	0.2	0.03	84	-16 ↓

↓ Decrease
↑ Increase
