# Supplementary material for: Deubiquitination of BES1 by UBP12/UBP13 promotes brassinosteroid signaling and plant growth
Source: Plant Commun. 2022 Jun 15;3(5):100348. doi: 10.1016/j.xplc.2022.100348 (PMC9483116; doi:10.1016/j.xplc.2022.100348)
Supplement: Document S2. Article plus supplemental information [file mmc2.pdf]

Plant Communications
Research Article

CellPress
Partner Journal

Deubiquitination of BES1 by UBP12/UBP13 promotes brassinosteroid signaling and plant growth

Su-Hyun Park[1], Jin Seo Jeong[1,2], Yu Zhou[1], Nur Fatimah Binte Mustafa[1] and Nam-Hai Chua[1,*]

[1]Temasek Life Sciences Laboratory, National University of Singapore, 1 Research Link, Singapore 117604, Singapore

[2]Present address: Wilmar International Limited, 28 Biopolis Road, Singapore 138568, Singapore

***Correspondence: Nam-Hai Chua (chua@rockefeller.edu)**

https://doi.org/10.1016/j.xplc.2022.100348

ABSTRACT

As a key transcription factor in the brassinosteroid (BR) signaling pathway, the activity and expression of BES1 (BRI1-EMS-SUPPRESSOR 1) are stringently regulated. BES1 degradation is mediated by ubiquitin-related 26S proteasomal and autophagy pathways, which attenuate and terminate BR signaling; however, the opposing deubiquitinases (DUBs) are still unknown. Here, we showed that the *ubp12-2w/13-3* double mutant phenocopies the BR-deficient dwarf mutant, suggesting that the two DUBs UBP12/UBP13 antagonize ubiquitin-mediated degradation to stabilize BES1. These two DUBs can trim tetraubiquitin with K46 and K63 linkages *in vitro*. UBP12/BES1 and UBP13/BES1 complexes are localized in both cytosol and nuclei. UBP12/13 can deubiquitinate polyubiquitinated BES1 *in vitro* and *in planta*, and UBP12 interacts with and deubiquitinates both inactive, phosphorylated BES1 and active, dephosphorylated BES1 *in vivo*. *UBP12* overexpression in *BES1^{OE}* plants significantly enhances cell elongation in hypocotyls and petioles and increases the ratio of leaf length to width compared with *BES1^{OE}* or *UBP12^{OE}* plants. Hypocotyl elongation and etiolation result from elevated BES1 levels because BES1 degradation is retarded by UBP12 in darkness or in light with BR. Protein degradation inhibitor experiments show that the majority of BES1 can be degraded by either the proteasomal or the autophagy pathway, but a minor BES1 fraction remains pathway specific. In conclusion, UBP12/UBP13 deubiquitinate BES1 to stabilize the latter as a positive regulator for BR responses.

Keywords: UBP12, UBP13, BES1, deubiquitination, brassinosteroid signaling

Park S.-H., Jeong J.S., Zhou Y., Binte Mustafa N.F., and Chua N.-H. (2022). Deubiquitination of BES1 by UBP12/UBP13 promotes brassinosteroid signaling and plant growth. Plant Comm. **3**, 100348.

INTRODUCTION

Brassinosteroids (BRs) play essential roles in diverse plant developmental processes, including hypocotyl and petiole elongation and leaf expansion (Nolan et al., 2020; Oh et al., 2020). Major components of the BR signaling pathway have been identified (Li et al., 2018; Lv and Li, 2020). The signaling cascade is initiated by the perception of BRs by the receptor-like kinase BRASSINOSTEROID-INSENSITIVE 1 (BRI1) on cell membranes, which phosphorylates and activates the co-receptor BRI1-ASSOCIATED KINASE 1 (BAK1). The signals are fine-tuned by inhibition of the glycogen synthase kinase 3-like kinase BRASSINOSTEROID-INSENSITIVE 2 (BIN2) and activation of the transcription factors BRI1-EMS-SUPPRESSOR 1 (BES1) and BRASSINAZOLE RESISTANT1 (BZR1). Eventually, BES1 and BZR1 directly regulate the transcription of thousands of downstream genes in response to various environmental conditions, such as light, drought, temperature, nutrients, and immune response toward pathogens (Sun et al., 2010; Yu et al., 2011; Li et al., 2018; Lv and Li, 2020; Nolan et al., 2020; Oh et al., 2020).

As key transcription factors for BR signaling, it is not surprising that levels of BES1/BZR1 proteins are finely regulated. Depending on external stimuli and during plant growth transition, various ubiquitin E3 ligases such as COP1 (Kim et al., 2014), MAX2 (Wang et al., 2013b), SINATs (Yang et al., 2017), and PUB40 (Kim et al., 2019) are known to polyubiquitinate and destabilize these factors by preparing them for 26S proteasomal degradation (Callis, 2014). Polyubiquitinated BES1 can also be degraded via selective autophagy (Nolan et al., 2017; Wang et al., 2021a). In general, polyubiquitinated proteins can be rescued from destruction by deubiquitinating enzymes (deubiquitinases [DUBs]), which remove mono- or polyubiquitin from target proteins, thereby increasing the stability of the latter. Such dynamic ubiquitination and deubiquitination reactions are important for fine-tuning signaling pathways. However,

Published by the Plant Communications Shanghai Editorial Office in association with Cell Press, an imprint of Elsevier Inc., on behalf of CSPB and CEMPS, CAS.

This is an open access article under the CC BY license (http://creativecommons.org/licenses/by/4.0/).

deubiquitination enzymes specific for BES1/BZR1 have not yet been identified.

Twenty-seven out of 64 *Arabidopsis* DUBs have been categorized as ubiquitin-specific proteases (UBPs), and all of them contain a conserved cysteine residue in their catalytic domain (Yan et al., 2000; March and Farrona, 2018). As members of a small UBP subfamily, UBP12 and UBP13 show 91% amino acid similarity and are functionally redundant. In addition to the catalytic domain, these two UBPs contain a conserved MATH (meprin and tumor necrosis factor receptor-associated factor homology) domain at their N terminus, which mediates protein–protein interactions (Cui et al., 2013; Eletr and Wilkinson, 2014). UBP12 and UBP13 have been implicated in diverse biological functions, including plant immune response (Ewan et al., 2011), onset of flowering (Cui et al., 2013), shade avoidance responses (Zhou et al., 2021), silencing of polycomb group protein genes (Derkacheva et al., 2016), jasmonic acid (JA) response (Jeong et al., 2017), root growth (An et al., 2018), leaf senescence under nitrogen deficiency (Park et al., 2019), circadian clock (Lee et al., 2019), and leaf size (Vanhaeren et al., 2020). Our previous study showed that *ubp12-2w/ubp13-3* double mutant plants (hereafter called *ubp12-2w/13-3*) are dwarf with small curved leaves and short petioles (Park et al., 2019). Because these phenotypes are typical of BR signaling and biosynthesis-deficient mutants, we hypothesized that UBP12/UBP13 might promote BR signaling. Here, we show that UBP12/UBP13 can indeed deubiquitinate BES1 to increase the stability of this transcription factor during plant cell elongation triggered by BR.

RESULTS

Mutant plants deficient in UBP12/UBP13 exhibit BR hyposensitivity

UBP12 and UBP13 contain the MATH domains and the catalytic UBP domain. The null double mutant *ubp12/ubp13* shows stunted growth and a pleiotropic phenotype. It is either infertile or rarely fertile, setting only a few seeds (Cui et al., 2013). Using Western blots, we analyzed protein levels of endogenous UBP12 in *ubp12-1*, *ubp12-2w*, *ubp13-1*, *ubp13-3*, and *ubp12-2w/13-3* and in *UBP12*- and *UBP13*-overexpressing plants (*UBP12^{OE}* and *UBP13^{OE}*) using an anti-UBP12 antibody. We also compared relative gene expression levels of *UBP12* and *UBP13* in these plants (Supplemental Figure 1). We confirmed that *ubp12-2w/13-3* produced the truncated protein UBP12ΔC, probably corresponding to a C-terminal truncation of UBP12, whereas *UBP12^{OE}* and *UBP13^{OE}* plants showed increased production of UBP12 and UBP13.

Preliminary observations showed that *ubp12-2w/13-3* plants phenocopied mutants deficient in BR biosynthesis or BR signaling. Wang et al. (2013a) previously used liquid chromatography-mass spectrometry and yeast-two hybrid experiments to show that UBP12 interacts with BZR1, which is functionally redundant with BES1. To explore a possible connection between UBP12/UBP13 and BR signaling, we analyzed cell elongation in *ubp12-2w/13-3* as well as plants overexpressing *UBP12* and *UBP13*. Compared with the wild type (WT), hypocotyl length, petiole length, and the ratio of leaf length to width were significantly decreased in *ubp12-2w/13-3* but slightly increased in *UBP12^{OE}* and *UBP13^{OE}* (Supplemental Figure 2A–2E). Expression levels of BR-responsive genes paralleled the observed phenotypes (Supplemental Figure 2F). In *ubp12-2w/13-3* plants, *CONSTITUTIVE PHOTOMORPHOGENIC DWARF* (*CPD*) and *C-22 hydrolase* (*DWF4*), whose expression is repressed by activated BR signaling, were expressed at higher levels compared with WT levels under normal conditions. By contrast, two BR-inducible genes, *SMALL AUXIN UP RNA 1 FROM ARABIDOPSIS THALIANA ECOTYPE COLUMBIA* (*SAUR-AC1*) and *ACC SYNTHASE 5* (*ACS5*), were expressed at a lower level under BR treatment. These results indicate that BR signaling is attenuated in *ubp12-2w/13-3* plants.

Because *ubp12-2w/13-3* showed reduced BR sensitivity, we compared its morphological and molecular phenotypes with those of plants with reduced *BES1* expression (*BES1-RNAi*; Yin et al., 2005). qRT–PCR analysis showed that *BES1* transcript levels in the *BES1-RNAi* plants were about 40% of WT values (Supplemental Figure 3). Figure 1A shows that 12-day-old seedlings of *ubp12-2w/13-3* exhibited a phenotype similar to that of *BES1-RNAi* seedlings. Hypocotyl elongation of *ubp12-2w/13-3* seedlings was hyposensitive to 24-epibrassinolide (eBL), an active form of BR, but hypersensitive to brassinazole (Brz), a BR biosynthesis inhibitor (Figure 1B and 1C). WT seedlings showed a 94% increase in hypocotyl length upon eBL treatment, whereas the comparable number in *ubp12-2w/13-3* was only 9%, very similar to that obtained with *BES1-RNAi*. With Brz treatment, hypocotyl lengths of WT seedlings were decreased by 61% compared with the non-treated group, but *ubp12-2w/13-3* seedlings showed a 73% inhibition of hypocotyl growth like *BES1-RNAi* seedlings. Time-course analysis of BR-inducible genes showed that *SAUR-AC1* and *ACS5* transcript levels were increased in WT but not in *ubp12-2w/13-3* under the same conditions. On the other hand, expression of the BR-repressed genes *CPD* and *DWF4* was 1.5–2 times higher in *ubp12-2w/13-3* compared with WT, but the expression levels declined rapidly in both genotypes during BR treatment. Control experiments showed no change in *BES1* and *UBP12* expression levels in response to BR treatment (Figure 2D). These results showed that the BR insensitivity could be attributed to UBP12 and UBP13 deficiency, suggesting a positive role for these two DUBs in BR responses.

UBP12/UBP13 bind to BES1 *in vitro*, and UBP12 interacts with both phosphorylated and dephosphorylated BES1 in plants

To investigate possible targets of UBP12/UBP13, we performed *in vitro* pull-down assays using purified proteins. Maltose-binding protein (MBP)-BES1-myc was pulled down by GST and GST-tagged full-length UBP12/UBP13 (Full) and by derivatives carrying the N- and/or C-terminal region (N and/or C). Figure 2A shows that UBP12 and UBP13 could directly interact with BES1. Further analysis of UBP12 derivatives showed that BES1 was specifically bound to the N-terminal region, which contains the MATH domain and the cysteine/histidine-containing catalytic domain. By dividing the BES1 protein into nine peptide fragments, we identified the BES1 fragment containing amino acids 99–164 as the region that interacts with UBP12 and UBP13 (Supplemental Figure 4).

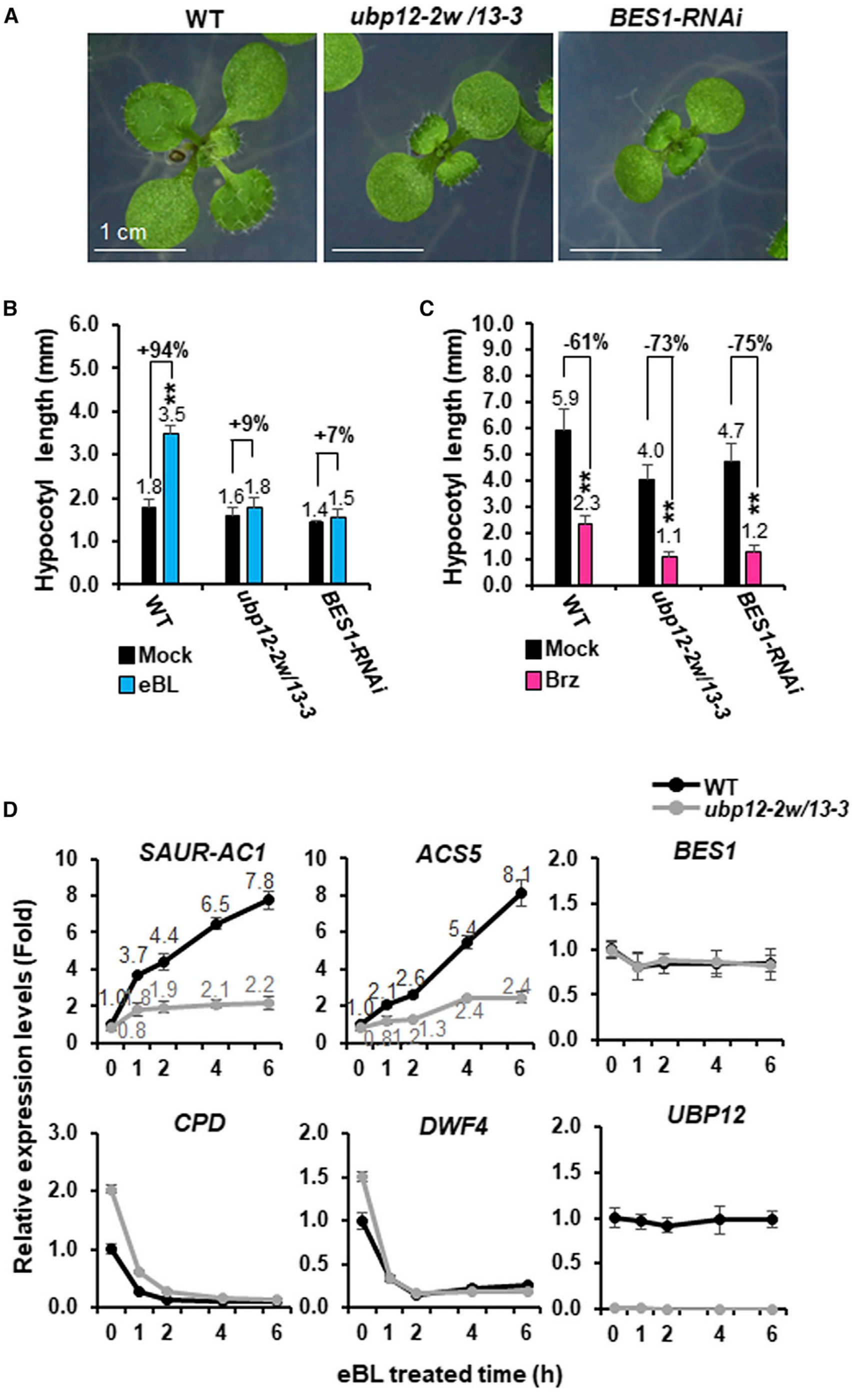

Figure 1. Phenotype comparison of the *ubp2-2w/13-3* double mutant and *BES1-RNAi*.

(A) *ubp12-2w/13-3* plants show growth retardation like *BES1-RNAi* plants. WT, *ubp12-2w/13-3*, and *BES1-RNAi* plants were grown on MS medium for 12 days under normal growth conditions. Scale bars correspond to 1 cm.

(B and C) Hypocotyl length (mm) of WT, *ubp12-2w/13-3*, and *BES1-RNAi* grown on MS medium containing 0.5 μM epi-brassinolide (eBL) under normal light (100 μmol m^{-2} s^{-1}) **(B)** and 0.8 μM brassinazole (Brz) under dim light (25 μmol m^{-2} s^{-1}) **(C)** for 7 days. Fifteen independent biological samples (n = 15) were analyzed. $^{*}P < 0.05$, $^{**}P < 0.01$ (two-tailed t-test).

(D) RNA expression dynamics of the BR-responsive genes *SAUR-AC1*, *ACS5*, *CPD*, and *DWF4* as well as *BES1* and *UBP12*. All samples were collected from 7-day-old seedlings of WT and *ubp12-2w/13-3* treated with 1 μM of eBL for 1, 2, 4, and 6 h. Expression levels of WT were set to 1. All qRT–PCR experiments were performed in triplicate. Average values of three independent biological samples (n = 3) are presented with standard deviations. *ACT2* expression levels were used as a control for normalization.

We first examined the UBP12–BES1 interaction in plants by semi-*in vivo* pull-down assays using *35S:BES1-myc* plants treated with or without eBL. pBES1-myc (phosphorylated BES1-myc) and BES1-myc (dephosphorylated BES1-myc) in protein extracts were pulled down with GST resin-bound UBP12. Both forms of BES1 showed strong interaction with GST-UBP12 but not with GST (Figure 2B). Under normal conditions, UBP12 interacted with both pBES1-myc and BES1-myc. The addition of BR facilitated BES1 dephosphorylation and accumulation of BES1-myc. Next, we performed co-immunoprecipitation (coIP) assays using extracts derived from *35S:BES1-myc/UBQ10:UBP12-HA* seedlings to examine the UBP12–BES1 interaction *in vivo*. pBES1-myc/BES1-myc and UBP12-HA proteins were immunoprecipitated with α-HA and α-myc antibodies, respectively, and the immunoprecipitates were analyzed by immunoblotting (Figure 2C). Figure 2C shows that UBP12-HA immunoprecipitates contained predominantly pBES1-myc and BES1-myc in the absence and presence of BR, respectively. Furthermore, BES1-myc immunoprecipitates also contained UBP12-HA. Bimolecular fluorescence complementation (BiFC) assays in tobacco leaves showed that BES1 interacted with full-length UBP12/UBP13 and their N-terminal derivatives, and the complex was detected in both the cytosol and nuclei under normal conditions (Figure 2D). BZR1, functionally redundant with BES1, also interacted with UBP12 and UBP13 *in vitro*, and these associations were confirmed *in vivo* by BiFC analysis (Supplemental Figure 5). Collectively, our results showed a direct association of UBP12/UBP13 with BES1 and its homolog BZR1. Moreover, UBP12/UBP13 strongly bind to active BES1 in plants treated with BR.

UBP12/UBP13 can deubiquitinate poly-ubiquitinated BES1 *in vitro*

The UBP12–BES1 interaction suggested that BES1 may be a substrate of UBP12. To examine this hypothesis, we explored whether UBP12 could function as a BES1 DUB *in vitro*. Because SINAT2 has been shown to polyubiquitinate BES1 (Yang et al., 2017), we used SINAT2 as an E3 ligase to produce

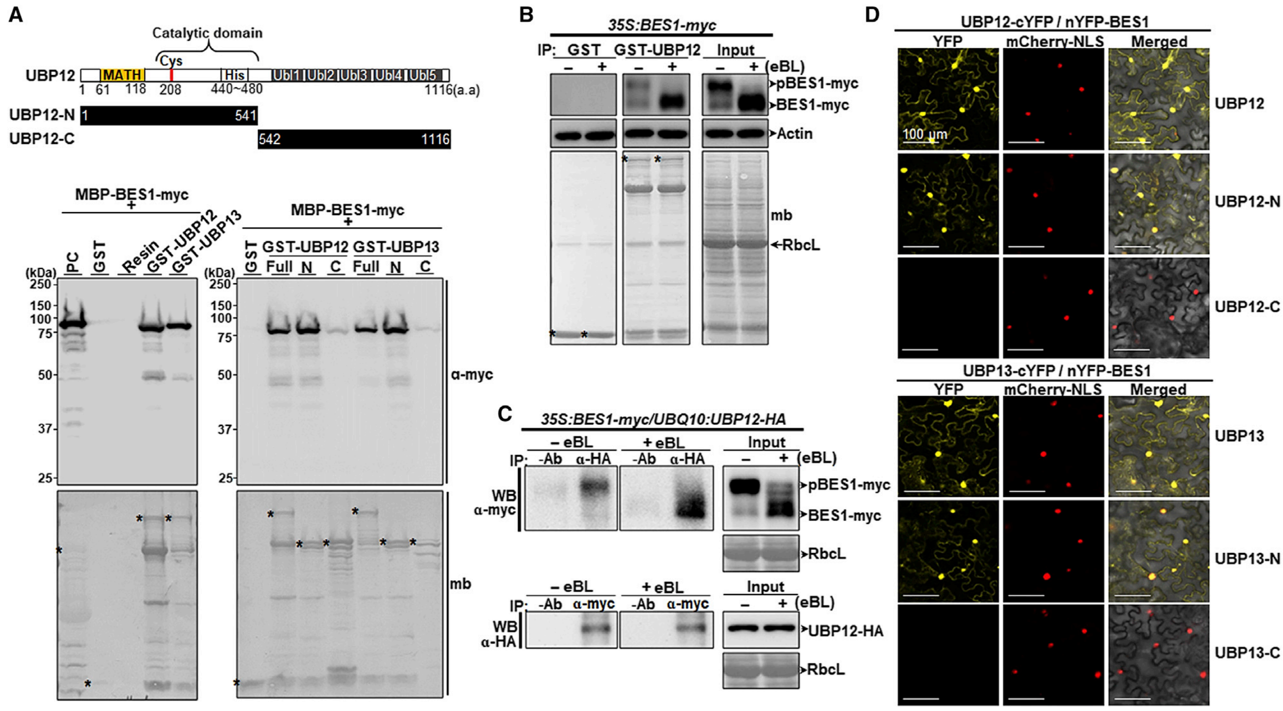

Figure 2. UBP12 and UBP13 interact with BES1 *in vitro* and *in vivo*.

(A) UBP12/UBP13 and BES1 interaction *in vitro*. MBP-BES1-myc was pulled down by GST and GST-tagged full-length UBP12/UBP13 (Full) and derivatives carrying the N- and/or C-terminal regions (N and/or C). The upper panel shows a schematic diagram of UBP12, and numbers indicate the amino acid positions. MATH, meprin and tumor necrosis factor receptor-associated factor homology domain; catalytic domain (called UBP in Supplemental Figure 1), ubiquitin-specific protease domain containing Cys and His motifs; Ubl, ubiquitin-like motifs. Bands were detected by immunoblots using anti-myc. Membrane (mb) was stained with Coomassie brilliant blue. About 2 μg of each protein was used in all experiments. The asterisks mark resin-bound bait proteins. MBP, maltose-binding protein; GST, glutathione-S-transferase; kDa, kilodalton. The positive control (PC) sample contained 50 ng MBP-BES1-myc.

(B) Semi-*in vivo* UBP12 and BES1-myc interaction. Seven-day-old *BES1-myc*-overexpressing plants (*35S:BES1-myc*) were treated with 1 μM epibrassinolide (eBL) for 6 h. BES1-myc from the total protein extracts was pulled down by resin-bound GST or GST-UBP12. The bands were detected by western blotting with anti-myc. GST, GST-UBP12, and total input proteins stained with Coomassie brilliant blue were used to show equivalent loading (mb).

(C) *In vivo* interaction of UBP12/UBP13 and phosphorylated/dephosphorylated BES1 (pBES1/BES1) in transgenic *Arabidopsis thaliana* (Col-0) plants expressing *35S:BES1-myc/UBPQ10:UBP12-HA.* Seven-day-old seedlings were treated with 50 μM MG132 in the presence or absence of 1 μM epibrassinolide (eBL) for 6 h. The pBES1-myc/BES1-myc and UBP12-HA proteins were immunoprecipitated with α-HA and α-myc antibodies, respectively. -Ab indicates the negative control without any antibodies. pBES1-myc/BES1-myc and UBP12-HA proteins in the immunoprecipitates were analyzed by immunoblots using α-myc and α-HA, respectively. Input proteins are shown. Levels of β-actin (Actin) and RbcL (the large subunit of ribulose-1,5-bisphosphate carboxylase) were used as loading controls.

(D) Interaction between UBP12/UBP13 and BES1 in tobacco (*Nicotiana benthamiana*) leaves assayed by bimolecular fluorescence complementation (BiFC). nYFP-BES1 was transiently co-expressed with UBP12-cYFP or UBP13-cYFP in leaves of *N. benthamiana* infiltrated with *Agrobacterium*. N termini of UBP12 and UBP13 were used as positive controls, whereas the C termini of the same proteins were used as negative controls. Localization is shown by merging YFP, mCherry-NLS (nuclear localization sequence), and DIC images (Merged). Confocal microscopy images were taken 3 days after infiltration. DIC, differential interference contrast. Scale bars correspond to 50 μm. Western blots and BiFC assays were analyzed in three independent experiments, and images of a representative set are shown.

polyubiquitinated BES1. GST-UBP12 and GST-UBP13 were treated with poly-ubiquitinated MBP-BES1-myc substrate for 0, 2, and 4 h. Figure 3A shows that UBP12 and UBP13 could indeed deubiquitinate polyubiquitinated BES1 *in vitro*. UBP12/UBP13 also interacted with SINAT2 *in vitro* and *in vivo*, but these two DUBs could not remove ubiquitin from polyubiquitinated SINAT2, indicating that this E3 ligase is not a target (Supplemental Figure 6).

In another experiment, full-length UBP12 and three different derivatives were co-incubated with MBP-BES1-myc substrate, enzymes E1, E2, and E3, and ubiquitin (Figure 3B). The three UBP12 derivatives were a partially inactive form of UBP12 in which cysteine 208 was substituted with serine (GST-UBP12_C208S) (Jeong et al., 2017), the N-terminal region of UBP12 (amino acids 1–541) (GST-UBP12-N), and the C-terminal region of UBP12 (amino acids 542–1116) (GST-UBP12-C) (Figure 2A). GST protein was used as a negative control (GST). The deubiquitinating enzymes and derivatives were added in increasing amounts. Figure 3B shows that UBP12 deubiquitination activity was significantly reduced by an active site mutation (C208S) in the enzyme. Interestingly, the N-terminal region of UBP12, which still contains the active site, did not show any enzyme activity, suggesting that the

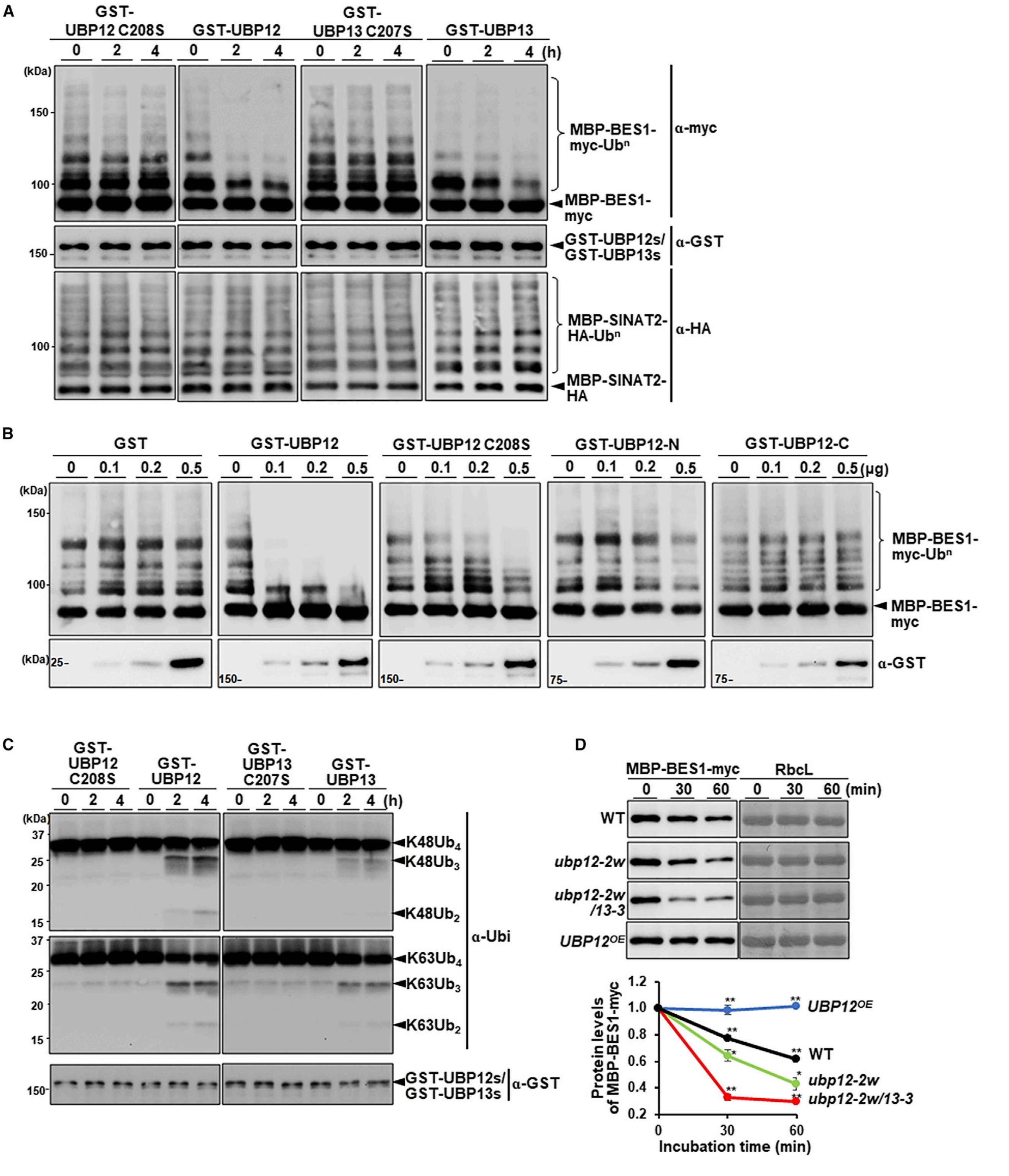

Figure 3. UBP12 stabilizes BES1 by deubiquitinating poly-ubiquitinated BES1.

(A) *In vitro* deubiquitination of polyubiquitinated MBP-BES1-myc and self-poly-ubiquitinated MBP-SINAT2-myc. Poly-ubiquitinated MBP-BES1-myc proteins were prepared by incubating MBP-BES1-myc with enzyme E1 (UBE1, human), E2 (UbcH6, human), and E3 (MBP-SINAT2-HA) using histidine-tagged ubiquitin as a donor (His-ubiquitin, 10.7 kDa). Poly-ubiquitinated MBP-BES1-myc (upper) and self-poly-ubiquitinated MBP-SINAT2-HA (lower) were treated with 500 ng GST-UBP12 and GST-UBP13 for 0, 2, and 4 h. GST-UBP12 C208S and GST-UBP13 C207S (Jeong et al., 2017) were used as negative controls. GST-UBP12 C208S, GST-UBP12, GST-UBP13 C207S, and GST-UBP13 were detected with anti-GST antibody.

(legend continued on next page)

C-terminal region, which comprises five ubiquitin-like (UBl) motifs (Figure 2A), may modulate the activity of DUB (Kim et al., 2016) through an allosteric effect.

Ubiquitin K63 linkages have been implicated in receptor cycling via endocytosis as well as in autophagy (Erpapazoglou et al., 2014; Romero-Barrios and Vert, 2018). A recent study reported that UBP13 can cleave the K63-linked polyubiquitin chain from BRI1 (Luo et al., 2022). Therefore, we examined the substrate specificity of UBP12/UBP13 using K48 and K63 tetra-ubiquitin as substrates. Figure 3C shows that UBP12/UBP13 actively cleave K48- as well as K63-linked ubiquitin and that the cysteine mutation at the active site blocked these activities.

We used a cell-free degradation assay to compare the fate of the recombinant protein MBP-BES1-myc in extracts prepared from plants of various genotypes: *ubp12-2w*, *ubp12-2w/13-3*, and *UBP12^{OE}*. MBP-BES1-myc was most rapidly degraded in *ubp12-2w/13-3* extracts compared with *ubp12-2w* and WT extracts, but MBP-BES1-myc levels were unaffected in extracts of *UBP12^{OE}* plants (Figure 3D). Taken together, our results show that the half-life of BES1 can be prolonged by UBP12 and UBP13, which remove ubiquitin from BES1.

UBP12 deubiquitinates both phosphorylated and dephosphorylated BES1 in plants

So far, our results showed that UBP12 bound to both pBES1 and BES1 in coIP assays and that UBP12/UBP13 could deubiquitinate polyubiquitinated BES1 *in vitro*. To see whether the phosphorylation status of BES1 would influence UBP12-mediated deubiquitination in plants, we performed *in vivo* ubiquitination assays (Figure 4). These assays were performed using WT, *BES1^{OE}*, *BES1^{OE}/UBP12^{OE}*, and *BES1^{OE}/ubp12-2w/13-3* treated with or without eBL. Ubiquitinated BES1-myc proteins were immunoprecipitated with TUBE (Tandem Ubiquitin Binding Entities) magnetic beads, and Ub_n-BES1-myc proteins in the immunoprecipitates were analyzed by immunoblots. Figure 4 shows that eBL treatment converted pBES1-myc into BES-myc. Both pBES1-myc and BES1-myc protein levels in *BES1^{OE}*, *BES1^{OE}/UBP12^{OE}*, and *BES1^{OE}/ubp12-2w/13-3* plants increased with increasing UBP12 levels. Polyubiquitinated-BES1-myc levels were lower in *BES1^{OE}/UBP12^{OE}* but higher in *BES1^{OE}/ubp12-2w/13-3* compared with those in *BES1^{OE}*. Together, these results support the notion that UBP12 is active toward both pBES1 and BES1, suggesting that the phosphorylation status of BES1 does not significantly affect the deubiquitination activity of UBP12. Because pBES1 is initially localized in the cytosol, our results also suggest that UBP12 is active in this cellular compartment as well.

UBP12 promotes plant growth by maintaining BES1 levels in BR signaling

We analyzed morphological and molecular phenotypes in WT, *BES1-RNAi*, *ubp12-2w/13-3*, *BES1-RNAi* in *ubp12-2w/13-3*, *BES1^{OE}*, *UBP12^{OE}*, *BES1^{OE}/UBP12^{OE}*, *BES1^{OE}/ubp12-2w/13-3*, and *UBP12^{OE}/BES1-RNAi* plants to assess whether UBP12/UBP13 could stabilize BES1 *in vivo*. We generated different plant genotypes by *Agrobacterium*-mediated transformation (Figure 5). *BES1-RNAi/ubp12-2w/13-3* was obtained by genetic crosses of *BES1-RNAi* and *ubp12-2w/13-3* plants. We used *UBP12* as a representative of UBP12/UBP13 because plants overexpressing *UBP12* alone exhibited a significant phenotype (Supplemental Figure 2). Figure 5 shows that hypocotyl and petiole lengths, the ratio of leaf length to width, and leaf area were markedly decreased in *BES1-RNAi*, *ubp12-2w/13-3*, and *BES1-RNAi/ubp12-2w/13-3* compared with the WT. The phenotype of *ubp12-2w/13-3* was similar to that of *BES1-RNAi* plants and was not exacerbated by the introduction of *BES1-RNAi*. This result suggests that UBP12 and UBP13 act in the same pathway as BES1 in BR signal transduction. By contrast, cell growth was significantly increased in *BES1^{OE}/UBP12^{OE}* compared with plants overexpressing either *BES1^{OE}* or *UBP12^{OE}* alone. Compared with the effects of *BES1$^{OE\ or\ UBP12OE\ alone\ on}$* lengths of various organs, overexpression of the two genes together (*BES1^{OE}/UBP12^{OE}*) produced an additive effect. In the double overexpression plants, there was an increase of 40%–50% in hypocotyl length, petiole length, and the ratio of leaf length to width and 37% in leaf area compared with the WT. However, overexpression of *BES1* in the *ubp12-2w/13-3* mutant background (*BES1^{OE}/ubp12-2w/13-3*) could not rescue the phenotype of *ubp12-2w/13*. *UBP12* overexpression in *BES1-RNAi* (*UBP12^{OE}/BES1-RNAi*) only partially restored hypocotyl and petiole length and leaf size, not even to WT levels (Figure 5A–5C; Supplemental Table 2). Similarly, overexpression of *UBP12* in *det2-1*, a BR-deficient mutant, only partially reversed the mutant dwarf phenotype. *UBP12^{OE}/det2-1* plants were much smaller compared with *UBP12^{OE}* plants and even with WT plants (Supplemental Figure 8). In both *det2-1* and *BES1-RNAi* plants, the overexpression effect of *UBP12^{OE}* was mild, whereas in the WT background (*UBP12^{OE}*),

(B) *In vitro* deubiquitination of MBP-BES1-myc by incubating enzymes E1, E2, and E3, His-ubiquitin with full-length UBP12, and three different derivatives. The deubiquitinating enzymes were added in increasing amounts (0, 0.1, 0.2, and 0.5 μg), and all the mixtures were incubated for 4 h at 30°C. Bands were detected by immunoblotting using α-myc antibody. The three derivatives of UBP12 were a partially inactive form of UBP12 in which cysteine 208 was substituted with serine (GST-UBP12 C208S) (Jeong et al., 2017), the N-terminal region of UBP12 (amino acids 1–541) (GST-UBP12-N), and the C-terminal region of UBP12 (amino acids 542–1116) (GST-UBP12-C), as shown in Figure 2A. GST protein was used as a negative control (GST). GST, GST-UBP12, GST-UBP12 C208S, GST-UBP12-N, and GST-UBP12-C were detected with anti-GST antibody.

(C) Deubiquitinase activity of UBP12 and UBP13 toward K48- or K63-linked ubiquitin *in vitro*. Recombinant human tetra-ubiquitin linked with K48 (upper panel) or K63 (lower panel) were used as substrates. The deubiquitinating enzymes (0.5 μg of each) and related active-site mutants were added to the mixtures and incubated for 2 and 4 h at 30°C. Bands were detected by immunoblotting using α-Ubi antibody. Anti-GST antibody was used to detect levels of UBP12/13 and related mutants.

(D) Cell-free degradation assays of recombinant MBP-BES1-myc protein in extracts prepared from WT, *ubp12-2w*, *ubp12-2w/13-3*, and *UBP12^{OE}* (*UBPQ10:UBP12-HA*) plants. The lower panel shows quantitative levels of MBP-BES1-myc. All mixtures were incubated at 30°C for 0, 30, and 60 min. RbcL levels were used as a loading control. Bands were detected by an immunoblotting assay using α-myc antibody. The lower graph shows quantitative results for the western blots. MBP, maltose-binding protein; GST, glutathione-S-transferase. All assays were performed with three independent experiments, and images from a representative set are shown. *$P < 0.05$, **$P < 0.01$ (two-tailed *t*-test).

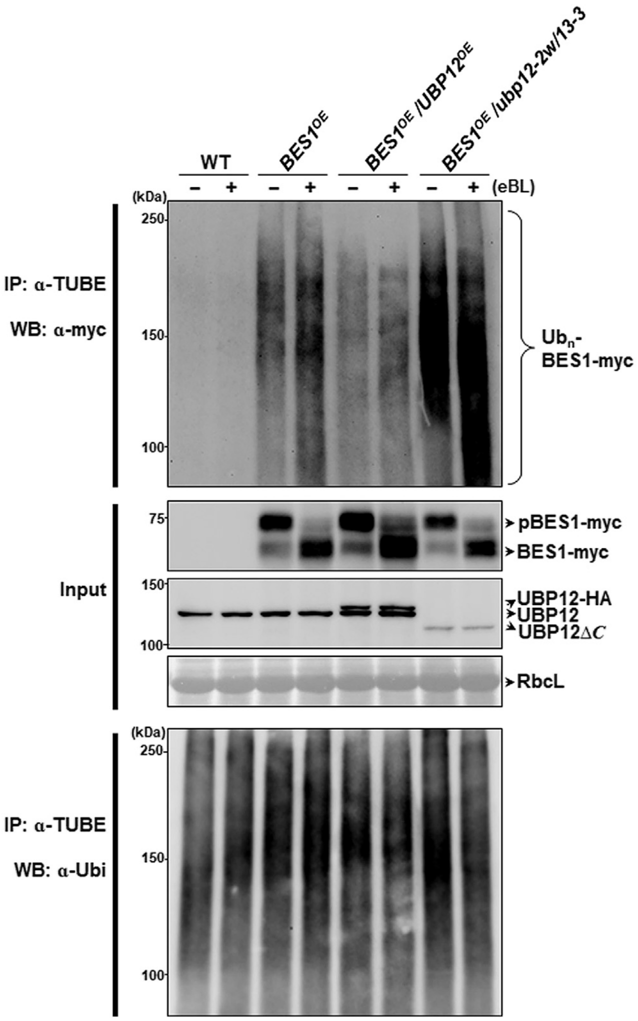

Figure 4. UBP12 deubiquitinates both phosphorylated and dephosphorylated BES1 in plants.

Ubiquitination assays were performed *in vivo* with 7-day-old seedlings of WT (Col-0), $BES1^{OE}$, $BES1^{OE}$/$UBP12^{OE}$, and $BES1^{OE}$/*ubp12-2w/13-3*, which were treated with 1 μM epi-brassinolide (eBL) for 6 h. Ubiquitinated BES1-myc proteins were immunoprecipitated with TUBE (Tandem Ubiquitin Binding Entities) magnetic beads. Ub_n-BES1-myc proteins in the immunoprecipitates were analyzed by immunoblotting using α-myc antibody. Western blotting with α-Ubi antibody was used as a positive control for the amount of immunoprecipitate in each reaction. Input pBES1-myc and BES1-myc and UBP12 proteins were detected by immunoblotting using α-myc and α-UBP12 antibodies, respectively. Levels of RbcL (the large subunit of ribulose-1,5-bisphosphate carboxylase) were used as a loading control. All assays were analyzed in three different experiments, and images of a representative set are shown.

overexpression of *UBP12* was able to promote growth resulting from BR signaling mediated by BES1 and its homologs (Supplemental Figure 2). These results indicate that the growth-promoting properties of UBP12 require BR and its major transcription factors BES1 and homologs.

The dominant mutant *bes1-D* harbors a point mutation (P233L) in the BES1 PEST domain. This mutant displays BR signal enhancement, including longer hypocotyls and petioles and curved leaf shapes. We found that bes1-D also interacted with UBP12/UBP13 *in vitro* (Supplemental Figure 7). Plants overexpressing *35S:bes1-D-YFP* in *ubp12-2w/13-3* exhibited only partial restoration of hypocotyl growth and reversal of dwarfism (Supplemental Figure 9), suggesting that the bes1-D protein is more unstable in the double mutant than in the WT. Moreover, *UBP12* overexpression promoted the growth of *bes1-D* plants, resulting in longer hypocotyls and petiole lengths and larger leaf sizes. $UBP12^{OE}$/*bes1-D* seedlings also showed hyponasty due to greater growth of the lower petiole surface, resulting in a narrower angle between two leaves compared with *bes1-D* seedlings (Supplemental Figure 10). These results suggest that UBP12 can stabilize bes1-D protein as well and that UBP12/UBP13 regulate BES1 in the same BR signaling process.

We analyzed transcript levels of genes induced or repressed by BR hormone using qRT–PCR (Figure 5D). BR-induced *SAUR-AC1* and *ACS5* transcript levels were markedly increased in $BES1^{OE}$/$UBP12^{OE}$ compared with the WT. Transcript levels of target genes in the double overexpressors were higher than those in the single overexpressors $BES1^{OE}$ or $UBP12^{OE}$. Conversely, *SAUR-AC1* and *ACS5* transcript levels were decreased in *BES1-RNAi*, *ubp12-2w/13-3*, *BES1-RNAi/ubp12-2w/13-3*, $BES1^{OE}$/*ubp12-2w/13-3*, and $UBP12^{OE}$/*BES1-RNAi* plants, which displayed BR-deficient phenotypes under eBL. The BR-repressed genes *CPD* and *DWF4* showed notably lower expression in $BES1^{OE}$/$UBP12^{OE}$ under eBL. By contrast, expression of these genes was increased in genotypes such as *BES1-RNAi*, *ubp12-2w/13-3*, *BES1-RNAi* in *ubp12-2w/13-3*, $BES1^{OE}$/*ubp12-2w/13-3*, and $UBP12^{OE}$/*BES1-RNAi* that showed a BR-deficient phenotype in the untreated mock condition, but the relative expression levels decreased rapidly under eBL (Figure 5D). These molecular phenotypes were consistent with the changes in hypocotyl length and the ratio of leaf length to width.

Plants of various genotypes were grown in an eBL-containing medium, and hypocotyl length was measured to investigate the functions of UBP12 and UBP13 in plant growth under the influence of BR hormone (Figure 5E). Compared with untreated controls, BR treatment increased WT hypocotyl length by 102%; the corresponding values for $BES1^{OE}$ and $UBP12^{OE}$ plants were 114%–115% and 135%–138%, respectively. The effect of *BES1* and *UBP12* double overexpression was almost additive, producing an increase of 151%–155% in hypocotyl length. By contrast, under the same conditions, hypocotyl lengths of *BES1-RNAi*, *ubp12-2w/13-3*, *BES1-RNAi/ubp12-2w/13-3*, and $BES1^{OE}$/*ubp12-2w/13-3* seedlings increased by only 32%–37%. These results are consistent with the phenotypes and BR-responsive gene expression observed in Figure 5A–5D. Our collective evidence indicates that UBP12 and UBP13 promote plant growth by maintaining BES1 levels in BR signaling.

UBP12 stabilizes BES1 protein under BR treatment and/or dark conditions

To investigate the *in vivo* relationship between BES1 protein levels and UBP12, we analyzed pBES1 and BES1 levels in mutants and transgenic plants with varying UBP12 levels. $BES1^{OE}$, $BES1^{OE}$/$UBP12^{OE}$, and $BES1^{OE}$/*ubp12-2w/13-3* plants were treated with or without eBL and with or without MG132, which blocks protein degradation (Figure 5F). Control experiments showed that *BES1* transcript levels were comparable among

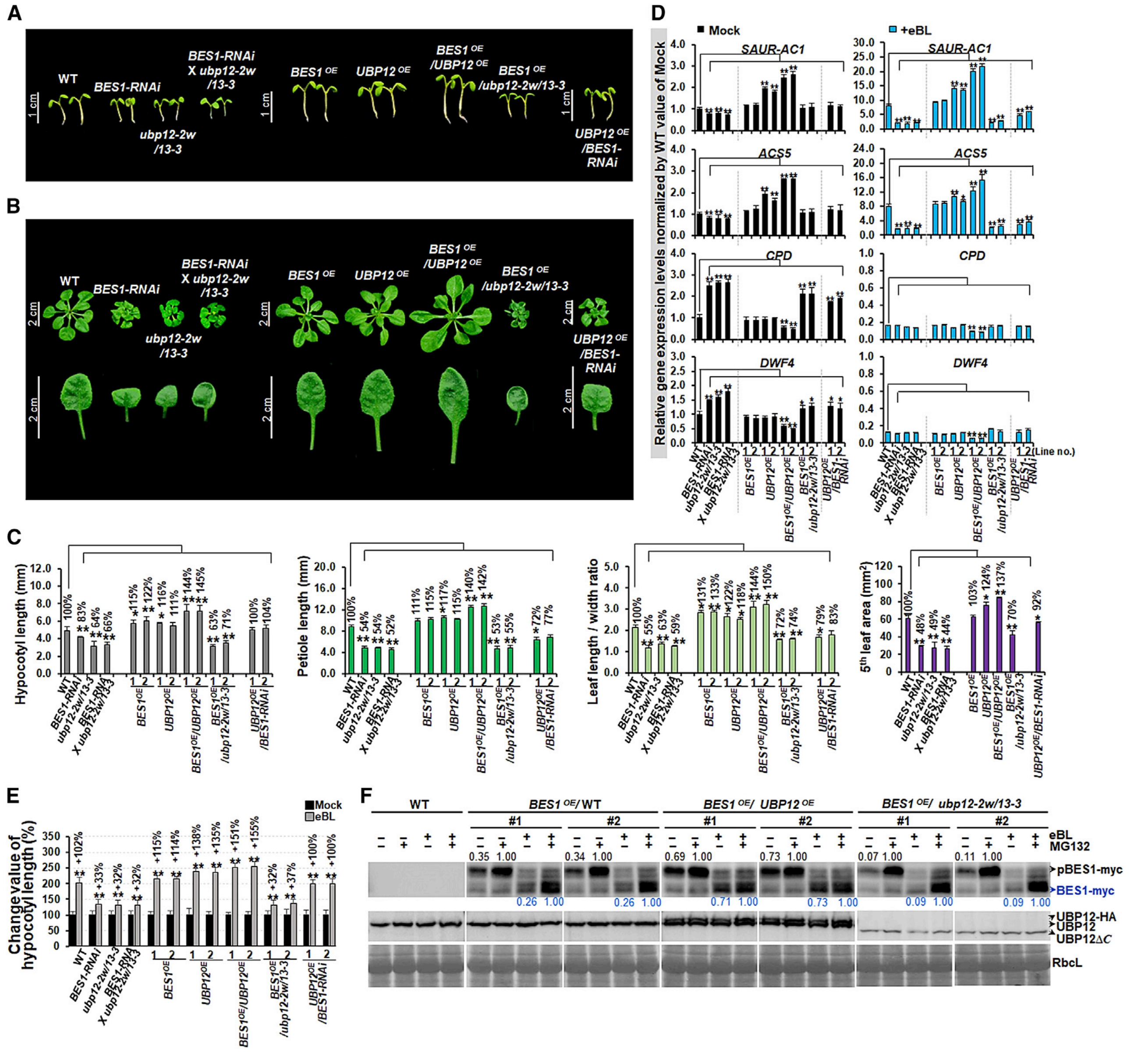

Figure 5. UBP12 promotes plant growth by maintaining BES1 levels in BR signaling.

Analysis of morphological and molecular phenotypes of various genotypes: WT, *BES1-RNAi*, *ubp12-2w/13-3*, *BES1-RNAi* in *ubp12-2w/13-3*, $BES1^{OE}$, $UBP12^{OE}$, $BES1^{OE}/UBP12^{OE}$, $BES1^{OE}$/*ubp12-2w/13-3*, and $UBP12^{OE}$/*BES1-RNAi*.

(A) Hypocotyl growth of 7-day-old seedlings grown under dim light (25 μmol m^{-2} s^{-1}). Scale bars correspond to 1 cm.

(B) Phenotypes of various genotypes grown on ½ MS medium for 3 weeks (upper) and the corresponding fifth leaves of the plants (lower). Scale bars correspond to 2 cm.

(C) Hypocotyl length measurements of plants in **(A)** and petiole length, leaf length/width ratio, and leaf area (mm^2) of the fifth leaves shown in **(B)**. Fifteen independent biological samples (n = 15) were obtained, and images from one representative experiment are shown. *$P < 0.05$, **$P < 0.01$ (two-tailed t-test).

(D) Transcript levels of BR-responsive genes were analyzed in 7-day-old seedlings of various genotypes treated with 1 μM eBL for 6 h using qRT–PCR. Expression levels of mock-treated WT plants were set to 1.

(E) Hypocotyl lengths of seedlings treated with BR. Seedlings were grown on MS medium containing 0.5 μM eBL for 7 days under normal conditions. Fifteen independent biological samples (n = 15) were obtained, and images from one representative experiment are shown. *$P < 0.05$, **$P < 0.01$ (two-tailed t-test).

(F) BES1 and UBP12 protein levels under BR treatment. MG132 (20 μM) was added to 7-day-old seedlings of $BES1^{OE}$/WT, $BES1^{OE}/UBP12^{OE}$, and $BES1^{OE}$/*ubp12-2w/13-3* (+MG132), and an equivalent amount of DMSO was added to control seedlings (−MG132). The two groups of seedlings were immediately treated with and without 1 μM eBL and then incubated for 6 h. WT was used as a negative control. Levels of the phosphorylated form

(legend continued on next page)

these three genotypes (Supplemental Figure 11). Relative levels of pBES1-myc and BES1-myc were normalized using RbcL as a loading control, and the gel bands corresponding to these forms are marked on the figure. Under normal conditions, pBES1-myc values were 0.34–0.38, 0.69–0.73, and 0.07–0.11 in *BES1^{OE}*, *BES1^{OE}/UBP12^{OE}*, and *BES1^{OE}/ubp12-2w/13-3* plants, respectively; by contrast, upon eBL treatment, BES1-myc values were around 0.26, 0.71–0.73, and around 0.09 in the corresponding genotypes. However, in both cases, levels of both BES1 forms could be elevated to comparable values by MG132. This analysis confirmed that both pBES1 and BES1 protein levels were elevated by *UBP12* overexpression but diminished by *UBP12/UBP13* deficiency, supporting the view that UBP12 stabilizes both forms of BES1 protein *in vivo*.

Previous studies have shown that BES1 is regulated by different E3 ligases depending on growth conditions: SINATs operate in the light (Yang et al., 2017), whereas COP1 is active in darkness (Kim et al., 2014). These findings led us to explore the possible regulation of BES1 by UBP12 under various growth conditions. In the light, *BES1^{OE}* showed a major band, pBES1-myc, and a minor band, BES1-myc, at the beginning of the experiment (0-h time point). In the presence of cycloheximide, which blocks new protein synthesis, levels of both BES1 forms decreased with time, indicating instability. Comparative experiments showed that protein decay was decreased by UBP12 overexpression (*BES1^{OE}/UBP12^{OE}*) but increased in the double mutant background (*BES1^{OE}/ubp12/2w/13-3*). eBL treatment of *BES1^{OE}* seedlings in the light caused dephosphorylation of pBES1-myc and the corresponding accumulation of active BES1-myc. BES1-myc was also the predominant species in etiolated *BES1^{OE}* seedlings (Figure 6A). No significant differences were detected for BES1-myc levels among these three genotypes at the beginning point. On the other hand, in the light with eBL and in darkness, BES1-myc levels decreased much faster in *BES1^{OE}/ubp12/2w/13-3* but were more stable in *BES1^{OE}/UBP12^{OE}* compared with *BES1^{OE}* (Figure 6A). Hypocotyl lengths of 7-day-old seedlings of *BES1^{OE}*, *BES1^{OE}/UBP12^{OE}*, and *BES1^{OE}/ubp12-2w/13-3* grown under continuous dark conditions were consistent with BES1 protein stability (Figure 6B). These results show that UBP12 functions to stabilize both pBES1 and BES1 under normal light conditions, but it also prevents BES1 from being degraded when BR signaling is stimulated endogenously in darkness or by exogenous hormone application.

Because BES1 can also be degraded via the selective autophagy pathway (Nolan et al., 2017; Wang et al., 2021a) we tested the effect of E64d, an autophagosome-specific inhibitor, on pBES1 and BES1 levels. Figure 6C shows that, similar to the results of MG132 treatment, E64d also increased pBES1/BES1 levels in mock-treated samples but not to the extent obtained with MG132. If the two degradative pathways process different populations of pBES1/BES1 we would expect an additive effect of the two inhibitors. We found only a moderate increase in pBES1/BES1 levels when the two inhibitors were added together, suggesting that the majority of pBES1/BES1 can be processed by either pathway and that only a minor fraction of pBES1/BES1 is degraded by specific pathways. Notwithstanding differences in the initial levels of pBES1/BES1 in *BES1^{OE}/UBP12^{OE}* and *BES1^{OE}/ubp12-2w/13-3*, similar responses to MG132 and E64d were seen in mock-treated seedlings of these samples. We also checked the regulatory function of UBP12 with respect to pBES1/BES1 proteins when BR signaling was activated by eBL and when BR biosynthesis was blocked by Brz. Irrespective of genotype, eBL addition converted inactive pBES1 to active BES1 compared with the mock-treated samples, whereas the opposite result was seen with the Brz inhibitor, although the effects were moderate. In these samples, both MG132 and E64d inhibited pBES1 and BES1 protein degradation, and pBES1/BES1 levels were obtained when the two inhibitors were added together. Collectively, these results suggest that UBP12 can stabilize pBES1 and BES1 by rescuing them from degradation by the 26S proteasomal as well as the autophagic pathway in the presence or absence of BR.

DISCUSSION

During the last decade, at least four different ubiquitin E3 ligases have been shown to downregulate BES1/BZR1 by polyubiquitination (Li et al., 2018; Lv and Li, 2020). In the dark, COP1 facilitates the degradation of phosphorylated BZR1, and skotomorphogenesis is induced with an increase in dephosphorylated BZR1 levels (Kim et al., 2014). In the light, on the other hand, SINAT E3 ligases destabilize BES1 to regulate BR-induced plant growth (Yang et al., 2017). The F-box protein MAX2 marks BES1 for destruction to control the number of shoot branches (Wang et al., 2013b), and in root tissues, the instability of phosphorylated BZR1 is promoted by PUB40 (Kim et al., 2019). In all these cases, BES1/BZR1 polyubiquitinated by E3 ligases are destroyed primarily via 26S proteasomes whose activity is blocked by MG132. In addition, particularly under stress conditions, polyubiquitinated BES1/BZR1 can also be removed by selective autophagy, which is sensitive to E64d (Nolan et al., 2017; Wang et al., 2021a). Further complexity may arise through cross-talk between the two pathways (Marshall et al., 2015; Zientara-Rytter and Subramani, 2019). We found that BES1 levels can be elevated by MG132 and also by E64d, although to a lesser extent (Figure 6C). The moderate synergistic effect of the two inhibitors suggests that the major fraction of polyubiquitinated BES1 can be redundantly degraded by either pathway, although a minor fraction remains pathway specific.

Because ubiquitination is a reversible process, it is not surprising to expect the participation of DUBs in BR signaling. However, nothing is known about the DUBs that counteract the action of these ubiquitin E3 ligases. Here, we show that UBP12/UBP13 can remove polyubiquitin chains from BES1 and rescue the latter from destruction. The following lines of evidence support our notion that UBP12/UBP13 are positive regulators of BR signaling: (1) UBP12/UBP13 can directly interact with

(pBES1-myc) and dephosphorylated form (BES1-myc) were analyzed using an anti-myc antibody. The transgene-derived UBP12-HA, the endogenous UBP12, and the truncated-UBP12 proteins were detected with anti-UBP12 antibodies (produced by rabbits immunized with an N-terminal UBP12 fragment). To determine the relative protein levels (indicated by numbers) of pBES1-myc and BES1-myc, the related protein level of each sample treated with MG132 was set to 1. RbcL levels were used as a loading control and for normalization. All assays were performed with three independent experiments, and images from a representative set are shown.

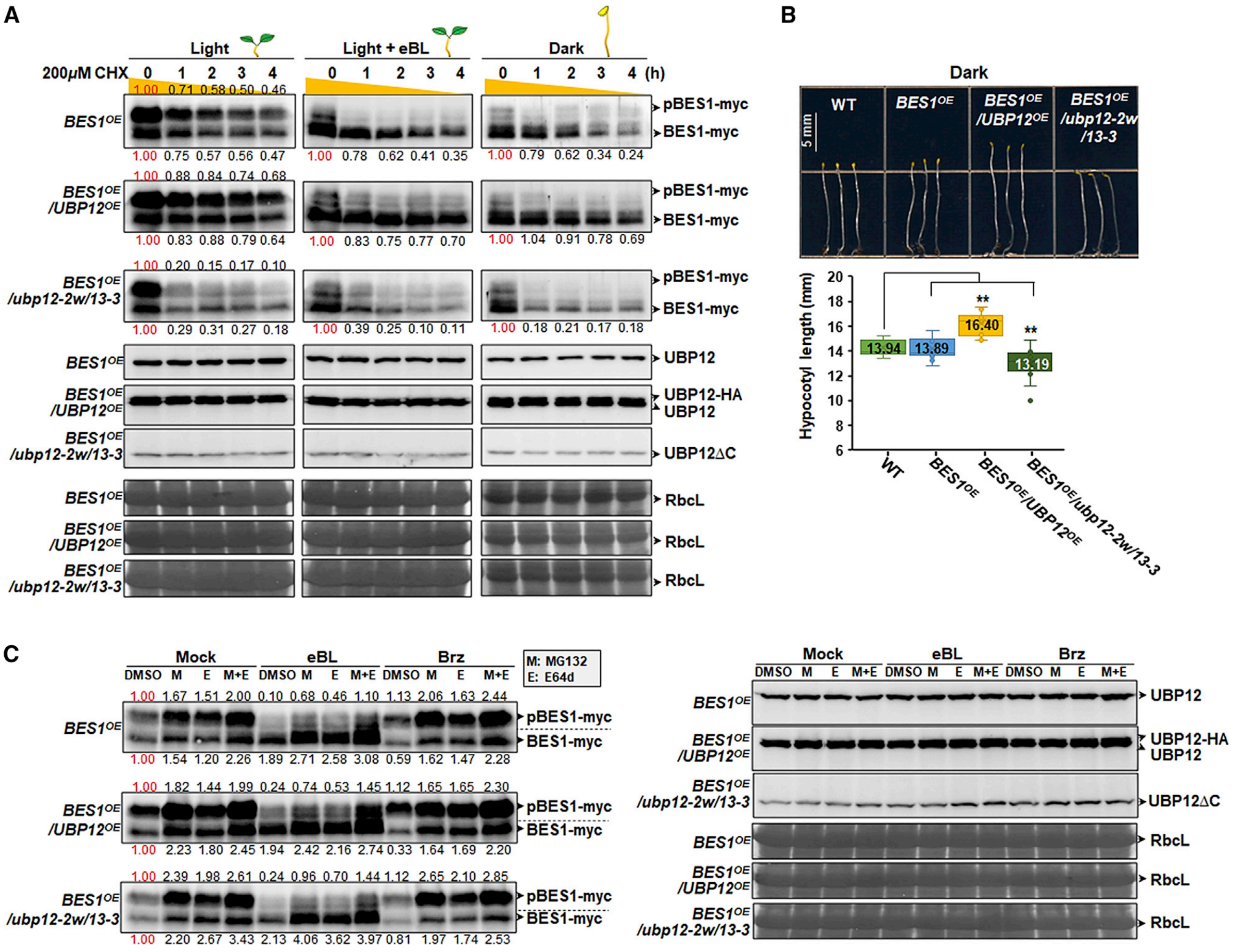

Figure 6. UBP12 stabilizes BES1 protein under BR signaling and dark conditions.

(A) Time course of BES1 protein levels after cycloheximide (CHX) treatment. Seven-day-old seedlings of *BES1*^{OE}/WT, *BES1*^{OE}/*UBP12*^{OE}, and *BES1*^{OE}/*ubp12-2w/13-3* grown under normal conditions (16 h light/8 h dark) were used. After 12 h of light, the seedlings were divided into two groups. For experiments in the dark, seedlings grown in continuous dark conditions were treated with 20 μM MG132 for 16 h in darkness. After rinsing with water, seedlings were treated with 200 μM CHX, and samples were taken hourly for 4 h. New protein synthesis in the treated seedlings was blocked by CHX, and levels of the phosphorylated form (pBES1-myc) and dephosphorylated form (BES1-myc) were analyzed by immunoblotting using an anti-myc antibody. The transgene-derived UBP12-HA, the endogenous UBP12, and the truncated-UBP12 (UBP12⊿C) proteins were detected with anti-UBP12 antibodies (produced by rabbits immunized with an N-terminal UBP12 fragment). To determine the relative protein levels (indicated by numbers) of pBES1-myc and BES1-myc, the related protein level at 0 h was set to 1. RbcL levels were used as a loading control and for normalization. All assays were analyzed in three different experiments, and images of a representative set are shown.

(B) Hypocotyl growth of 7-day-old seedlings grown under dark conditions. Scale bar corresponds to 5 mm (left panel). For hypocotyl length measurements of seedlings (right panel), 15 independent biological samples (*n* = 15) were obtained, and images from one representative experiment are shown. Average values are marked on the plot. $^{*}P < 0.05$, $^{**}P < 0.01$ (two-tailed *t*-test).

(C) DMSO, 20 μM MG132 (M), 20 μM E64d (E), and MG132 plus E64d (M+E) were added to 7-day-old seedlings of *BES1*^{OE}/WT, *BES1*^{OE}/*UBP12*^{OE}, and *BES1*^{OE}/*ubp12-2w/13-3* grown under normal conditions (16 h light/8 h dark), and the treated seedlings were divided into three groups. The groups were separately incubated with control (Mock), 1 μM eBL, and 5 μM Brz in the light for 6 h. Immunoblots were analyzed as in **(A)**.

BES1 *in vitro*. Wang et al. (2013a) previously used liquid chromatography-mass spectrometry and yeast-two hybrid experiments to show that UBP12 interacts with BZR1, which is functionally redundant with BES1. Our results confirm and extend their observations on BZR1. (2) UBP12/UBP13 can deubiquitinate polyubiquitinated BES1, and this DUB activity is dependent on C208 of the UBP12 catalytic domain. (3) UBP12/UBP13 bind to both phosphorylated and dephosphorylated BES1 *in vivo*, and the UBP12/BES1 and UBP13/BES1 complexes are detected in both the cytosol and nuclei. (4) In the light, both forms of BES1 are degraded more rapidly in the *ubp12-2w/13-3* double mutant but more slowly in UBP12/UBP13 overexpression plants compared with the WT. (5) In the light with BR and in darkness, degradation of the predominating BES1 is protected by UBP12/UBP13. (6) Growth phenotypes and BR-responsive gene expression in *ubp12-2w/13-3* and *UBP12* overexpression plants are correlated with their BES1 protein levels. Taken together, our results show that UBP12 and UBP13 can directly associate with BES1 and prevent its destruction, thereby promoting BR signaling.

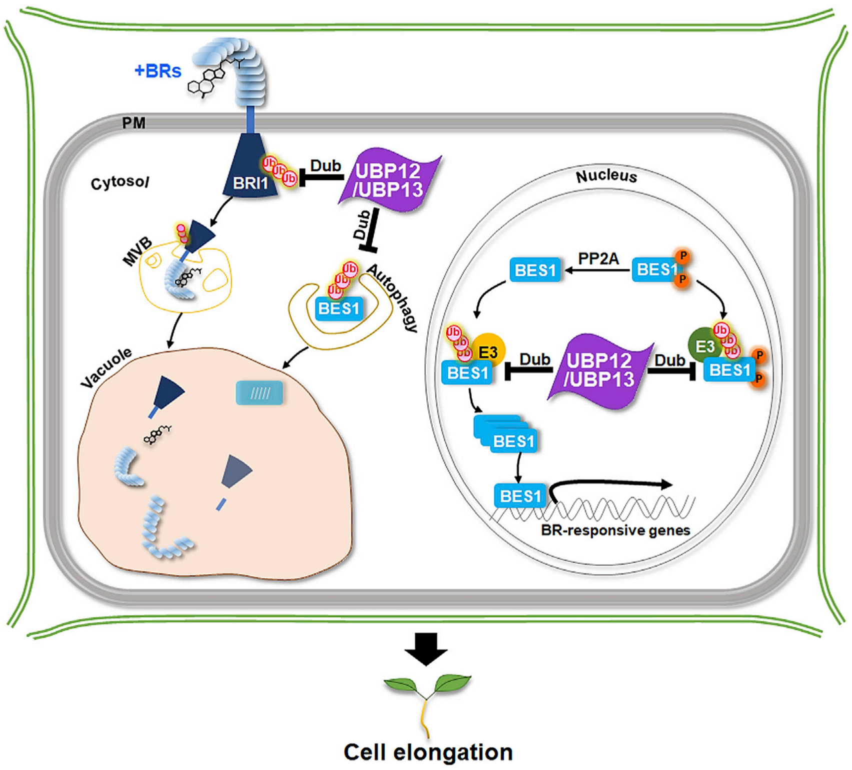

Figure 7. A working model for the positive regulatory roles of UBP12/UBP13 during BR signaling.

BR signaling triggers import of pBES1 (the inactive form) into the nucleus where it becomes dephosphorylated by nuclear-localized PP2A (Wang et al., 2021b). Both forms of BES1 are polyubiquitinated by various E3 ligases (SINATs, COP1, etc.), targeting them for degradation by 26S proteasomes. However, polyubiquitinated BES1 and pBES1 can be rescued from destruction by the deubiquitinases UBP12/UBP13, which stabilize and increase cytosolic and nuclear BES1 and pBES1 levels. The active BES1 transcription factor activates downstream genes *SAUR-AC1* and *ASC5*, resulting in cell elongation. Concurrently, BES1 represses the expression of the BR biosynthetic genes *CPD* and *DWF4*. Recently, Luo et al. (2022) reported that UBP12/UBP13 can directly deubiquitinate the BRI1 protein. Deubiquitination of BRI1 limits vacuole targeting of BRI1 through the multivesicular body (MVB) pathway and degradation. Polyubiquitinated BES1/BZR1 can also be removed by selective autophagy (Nolan et al., 2017; Wang et al., 2021a). Consequently, UBP12/UBP13 regulate BR signaling in at least two sites: the top and the bottom of the pathway.

Our findings on the relationship between UBP12 and pBES1/BES1 presented here can be summarized by the simple working model shown in Figure 7. Upon BR signaling, pBES1 is imported into the nucleus, where it is dephosphorylated by PP2A to give the active BES1 (Wang et al., 2021b). Both forms of BES1 are marked for proteasomal degradation by a number of E3 ligases (Wang et al., 2013b; Kim et al., 2014, 2019; Yang et al., 2017). UBP12/UBP13 can oppose this process and increase BES1 stability by deubiquitination. The elevated BES1 levels activate or depress downstream gene expression. Luo et al. (2022) recently reported that UBP12/UBP13 could remove K63-linked polyubiquitin chains from the BRI1 protein, thereby limiting endocytosis-mediated degradation of BRI1. Here, we show that UBP12 can stabilize pBES1 and BES1 targeted for degradation via 26S proteasomes as well as by autophagy in both the presence and absence of BR. Under Brz treatment, the BR biosynthesis inhibitor UBP12 can also rescue BES1 proteins from destruction (Figure 6C). These results pertaining to the regulation of BES1 by UBP12/UBP13 in the nucleus and cytosol are related to but clearly independent of BRI1 regulation at the plasma membrane. Therefore, UBP12/UBP13 can regulate BR signaling not only at the signal perception level (BRI1) but also at the transcription factor (BES1/BZR1) level. The ability to fine-tune both at the top and the bottom of the signaling pathway allows for more flexible and rapid responses to changing BR levels.

Our *in vitro* experiments showed that UBP12 and UBP13 could associate with the amino acid 99–164 region of BES1, which is enriched in serine/threonine residues. The region is close to the phosphorylation domain that binds the 14-3-3 protein (Gampala et al., 2007; Ryu et al., 2007) (Supplemental Figure 4). The phosphorylation domain of BZR1 is required for its nuclear export triggered by the BIN2 kinase (Li et al., 2001; Li and Nam, 2002; House et al., 2003). Our observation that UBP12 binds to active BES1 under BR treatment suggests that the UBP12/BES1 complex may shield the bound BES1 from phosphorylation by BIN2 kinase. In this regard, UBP12 and UBP13 are also expected to oppose the action of 14-3-3 protein and BIN2, both of which are negative regulators of BR signaling.

Although plant DUBs have not been as extensively investigated as ubiquitin E3 ligases, nonetheless, results to date show that these enzymes play crucial roles in plant growth and development (Majumdar and Nath, 2020). The functionally redundant UBP12 and UBP13 are expressed at a reasonably high level throughout the plant life cycle. The null double mutant *ubp12/ubp13* is lethal (Cui et al., 2013; An et al., 2018), indicating that the two encoded DUBs are essential for plant survival. In the *ubp12-2w/13-3* double mutant, the *UBP12* coding sequence is interrupted at its 3′ end by a T-DNA insertion. The resulting UBP12ΔC form, which is expressed from the truncated gene and maintained at a low level, can confer partial function (Supplemental Figure 1), allowing the survival of *ubp12-2w/13-3*. Plants of this double mutant are dwarf and partially fertile, producing only a limited number of seeds (Cui et al., 2013) and displaying a BR-deficient phenotype. UBP12 and UBP13 have been shown to bind to the latent peptidases DA1, DAR1, and DAR2 *in vivo* and to inactivate protease function by removing ubiquitin (Vanhaeren et al., 2020). DA1, DAR1, and DAR2 act as negative regulators of leaf growth and are activated via multiple mono-ubiquitination by the E3 ligases BIG BROTHER and DA2. These latent peptidases inhibit plant growth by cleaving and degrading growth regulators such as UBP15, TCP14, TCP15, and TCP22 (Dong et al., 2017). As such, UBP12 and UBP13 can

induce plant growth by stabilizing BRI1 and BES1 as well as by eliminating the activity of DA1, DAR1, and DAR2 proteases, which restrain growth. These findings support a pivotal role for UBP12 and UBP13 in the promotion of plant growth and development.

Given the reported roles of UBP12/UBP13 in several signaling pathways, we can surmise that the dwarf phenotype of the double mutant cannot be attributed solely to a deficiency in BR signaling. Our finding that the BR-deficient phenotype of *ubp12-2w/13-3* is only partially restored by overexpression of *bes1-D* supports this view (Supplemental Figure 9). In addition to BRs, gibberellins (GAs) and auxins are important phytohormones with broad functions in hypocotyl elongation and leaf and root growth. Mutants defective in GA or auxin signaling pathways all display a dwarf stature with reduced hypocotyl, rosette leaf, and root growth (Koornneef and van der Veen, 1980; Griffiths et al., 2006; Kasahara, 2016). In addition, cross-talk of BR with GA and auxin has been extensively reported (Ohri et al., 2019). Therefore, it is not surprising that UBP12/UBP13 should target key components of GA and auxin signaling pathways as well.

The *Arabidopsis* genome encodes more than 1400 ubiquitin E3 ligases (Vierstra, 2009; Wang and Deng, 2011) but only 64 potential DUBs (Isono and Nagel, 2014), suggesting that one DUB may oppose the action of several E3 ligases. Our observations that UBP12/UBP13 can counteract the action of PUB10 E3 ligase and NLA E3 ligase in JA signaling and nitrogen starvation-induced leaf senescence, respectively, support this notion (Jeong et al., 2017; Park et al., 2019). Because BR is also involved in accelerating leaf senescence during normal growth, it is likely that BES1 may also function along with UBP12/UBP13 in N-induced leaf senescence. We previously observed that *UBP12/UBP13* transcript levels increased threefold in response to N starvation (Park et al., 2019). However, we found here that *UBP12* transcript and protein levels did not change for 6 h under eBL treatment (Figures 1D and 5F); moreover, no change in protein levels was observed even under light and dark conditions (Figure 6). Whether UBP12/UBP13 activities can be regulated by post-translational events remains to be investigated.

In summary, during BR signaling, BES1 is polyubiquitinated by various E3 ligases and rapidly degraded via 26S proteasomes and/or autophagy, but polyubiquitinated BES1 can be rescued from destruction by polyubiquitin chain removal mediated by UBP12/UBP13. The delicate balance between BES1 degradation and stabilization controls the expression of downstream genes, such as *SAUR-AC1*, *ASC5*, *CPD*, and *DWF4*, thereby regulating cell growth (Figure 7). Such dynamic fine-tuning of BES1 protein levels provides plants with the flexibility to rapidly regulate plant tissue elongation in response to changes in diverse developmental cues. The positive regulation of plant growth mediated by UBP12/UBP13 can be exploited to improve crop plant yield.

MATERIALS AND METHODS

Plant materials and growth conditions

Arabidopsis thaliana Columbia ecotype (Col-0) was used as the WT. A number of T-DNA insertion mutant lines were used: *ubp12-1* (GABI_244E11), *ubp12-2w* (GABI_742C10), *ubp13-1* (SALK_128312), *ubp13-3* (SALK_132368), and *ubp12-2w/13-3* (Cui et al., 2013). $UBP12^{OE}$ and $UBP13^{OE}$ transgenic plants expressing *UBQ10:UBP12-HA* and *UBQ10:UBP13-HA*, respectively, were described by Jeong et al. (2017). Plants expressing *BES1-RNAi* were reported previously (Yin et al., 2005). Genetic crosses were performed to obtain the triple mutant *ubp12-2w/13-3/BES1-RNAi*. The *35S:BES1-myc* transgene was introduced by transformation into WT, $UBP12^{OE}$, and *ubp12-2w/13-3* to obtain $BES1^{OE}$, $BES1^{OE}/UBP12^{OE}$, and $BES1^{OE}$/*ubp12-2w/ubp13-3* plants, respectively. *BES1/RNAi* plants were transformed with *Agrobacterium* carrying *UBQ10:UBP12-HA* to obtain $UBP12^{OE}$/*BES1-RNAi*. The floral dipping method mediated by *Agrobacterium* strain GV3101 was used to generate all transgenic plants in this study (Clough and Bent, 1998; Zhang et al., 2006). Seeds were germinated on ½ MS solid medium (0.8% agar) containing 1% sucrose and adjusted to pH 5.7. Plants were grown under a long-day regime with 16 h light (100 μmol m^{-2} s^{-1}) and 8 h dark at 22°C for the indicated durations.

Plasmid DNA construction

Full-length *BES1* and *BZR1* coding sequences were amplified from cDNA of WT plants using RT–PCR. *35S:BES1-myc* was used to generate overexpressing plants, and *35S:nYFP-BES1* was produced for BiFC assays in tobacco. Derivatives of BES1 (ΔC1, ΔC2, ΔC3, ΔN1, ΔN2, ΔN3, M, M1, and M2) for *in vitro* pull-down assays were produced using the indicated primer pairs. Vectors to produce BES1 and BZR1 were constructed by fusing the appropriate cDNA sequences with DNA sequences encoding N-terminal MBP-tag and C-terminal 6x myc-tag. All constructs were generated by the Gateway cloning system with BP Clonase II Enzyme (Invitrogen, cat. no. 11789100) and LR Clonase II Enzyme (Invitrogen, cat. no. 11791100). Primers used for vector construction are listed in Supplemental Table 1.

Phenotype analysis

For hypocotyl length analysis, plants were grown under 16 h dim light (25 μmol m^{-2} s^{-1}) and 8 h dark at 22°C for the indicated period. Petiole lengths and leaf length/width ratio were measured using 3-week-old plants grown at 22°C under long-day conditions. Sixteen 10-day-old seedlings of each type were transferred onto a large Petri dish (*d* = 120 mm) containing new MS medium. There was sufficient space between seedlings to avoid interference with plant growth. Plants were then incubated under long-day conditions (16 h light/8 h dark; light intensity 100 μmol m^{-2} s^{-1}) for an additional 11 days, as shown in Figure 5 and Supplemental Figures 2C, 8B, 9B, and 10B. After taking images of plants, the hypocotyl length, petiole length, leaf length (the longest point), and leaf width (the widest point) were measured with ImageJ software.

qRT–PCR analysis

Total RNA isolated by TRIzol (Invitrogen, cat. no. 15596026) was treated with an RNeasy mini kit (QIAGEN, cat. no. 74104) and DNase I (QIAGEN, cat. no. 79254). cDNA was synthesized using a RevertAid First Strand cDNA Synthesis Kit (Thermo Scientific, cat. no. K1622) from 1 μg of total RNA. Quantitative real-time PCR was performed with the synthesized cDNA as a template and gene-specific primers for *SAUR-AC1*, *ACS5*, *CPD*, *DWF4*, *BES1*, *UBP12*, and *UBP13* using SsoAdvanced Universal SYBR Green Supermix (Bio-Rad, cat. no. 172–5275) and analyzed using a CFX96 Real-Time PCR system (Bio-Rad). All qRT–PCR experiments were repeated three times and normalized with *ACT2* expression levels. The average value of three independent biological replicates ($n = 3$) is presented with standard deviation (±SD). The qRT–PCR data were statistically compared with the control using a two-tailed *t*-test (*$P < 0.05$, **$P < 0.01$). Primers used in this study are listed in Supplemental Table 1.

In vitro pull-down assays

In vitro pull-down assays were performed as described previously (Park et al., 2019). pMAL-DC and pGEX-DC were transformed into *Escherichia*

coli strain BL21 (DE3) pLysS (Novagen, cat. no. 69451). Recombinant proteins were induced with 0.1 mM isopropyl-β-D-thiogalactoside at 22°C for 16 h and purified using amylose resin (New England Biolabs, cat. no. E8021) for the MBP tag or Glutathione Sepharose 4 Fast Flow (GE Healthcare, cat. no. 17-5132-01) for the GST tag. Recombinant proteins MBP-BES1-myc and MBP-BZR1-myc were individually incubated with GST resin-bound proteins: GST, GST-UBP12, GST-UBP13, GST-UBP12-N, GST-UBP12-C, GST-UBP13-N, and GST-UBP13-C. After washing with a wash buffer, the beads were boiled with a protein loading buffer. Pulled down proteins separated by SDS–PAGE were immunoblotted with anti-myc (Proteintech, cat. no. 16286-1-AP) or anti-MBP (Proteintech, cat. no. 15089-1-AP) antibody using the ECL Prime Western Blotting System (GE Healthcare, cat. no. RPN2232). Two micrograms of recombinant protein were used in all experiments. Pull-down assays for the truncated forms of MBP-BES1 with GST resin-bound protein GST-UBP12 and GST-UBP13 were performed using the same procedure. All assays were analyzed in three independent experiments.

In vivo coIP assay

Seven-day-old seedlings of *35S:BES1-myc/UBQ10:UBP12-HA* were incubated with 50 μM MG132 (Calbiochem, cat. no. 474790) in the presence or absence of 1 μM eBL (Sigma, cat. no. E1641) for 6 h at 22°C under light conditions. Treated plants were collected and homogenized with coIP buffer consisting of 50 mM Tris–HCl (pH 7.4), 100 mM NaCl, 0.2% Triton X-100, 1 mM DTT, and protease inhibitor cocktail (Roche, cat. no. 04693159001). An aliquot of the total protein was saved as an input sample. Total protein extracts were incubated with protein A-agarose for 1 h at 4°C to remove adventitious binding proteins. The antibodies anti-myc (Proteintech, cat. no. 60003-2-Ig), anti-HA (Santa Cruz, cat. no. SC-805), and anti-GFP (Santa Cruz, cat. no. SC-9996) were added to the treated protein extracts for 2 h at 4°C. The antibody/protein complex, which was retrieved by incubating with 20 μl of protein A-agarose for 2 h at 4°C, was washed with a coIP buffer 5 times. The co-immunoprecipitated proteins were separated by SDS–PAGE followed by immunoblotting assays with anti-HA (Santa Cruz, cat. no. SC-805), anti-myc (Proteintech, cat. no. 16286-1-AP), and anti-Actin (ProteinTech, cat. no. 66009-1-Ig) antibodies using the ECL Prime Western Blotting System (GE Healthcare, cat. no. RPN2232). All assays were repeated in three independent experiments.

Semi-*in vivo* pull-down assays

Seven-day-old seedlings overexpressing BES1-myc were grown under normal conditions, and seedlings were treated with or without 1 μM eBL (Sigma, cat. no. E1641) for 6 h. Total protein extracts were prepared from ground powders by resuspension in a protein extraction buffer containing 100 mM Tris–HCl (pH 7.5), 300 mM NaCl, 2 mM EDTA (pH 8.0), 1% Triton X-100, 10% glycerol, and protease inhibitor cocktail (Roche, cat. no. 04693159001) (Yang et al., 2017). Supernatants were pre-incubated with GST resin at 4°C for 1 h and then incubated with GST resin-bound GST and GST-UBP12 at 4°C for 2 h. After the beads were washed 5 times with a protein extraction buffer, the precipitated proteins were separated by SDS–PAGE and analyzed by immunoblotting with anti-myc antibody (Santa Cruz, cat. no. SC-9996). All assays were repeated in three independent experiments.

BiFC assays

Agrobacterium GV3101 strains carrying *35S:nYFP-BES1*, *35S:UBP12-cYFP*, *35S:mCherry-NLS*, and *35S:P19* were incubated at 28°C for 16 h. Harvested cells were treated with 150 μM acetosyringone (Aldrich, cat. no. D134406) at 25°C for 3 h after resuspending in 10 mM $MgCl_2$. The *Agrobacterium* cell mixture was infiltrated into leaves of 3-week-old tobacco plants (*Nicotiana benthamiana*). BiFC images were observed using an SP8 confocal scanning laser microscope (Leica) 3 days after infiltration. All assays were analyzed in three independent experiments.

Ubiquitination and deubiquitination assays *in vitro*

To obtain polyubiquitinated MBP-BES1-myc *in vitro*, 500 ng of MBP-BES1-myc was mixed with 55 nM of human UBE1 (E1; Boston Biochem, cat. no. E-304), 166 nM of human UbcH5b (E2; Boston Biochem, cat. no. E2-622), 500 ng of GST-SINAT2 (E3), and 2 μg of histidine-tagged ubiquitin (His-ubiquitin, 10.7 kDa) in 30 μl of a ubiquitination buffer (50 mM Tris [pH 7.4], 5 mM $MgCl_2$, 2 mM DTT, and 2 mM ATP). For *in vitro* autoubiquitination assays of the SINAT2 E3 enzyme, 500 ng of MBP-SINAT2-myc was mixed with the same amount of E1, E2, and His-ubiquitin in the ubiquitination buffer. All reaction mixes were incubated for 4 h at 30°C. For deubiquitination assays, the recombinant proteins GST-UBP12 and GST-UBP13 were purified from *E. coli* after induction with IPTG (isopropyl-β-D-thiogalactoside). Purified GST-UBP12 and GST-UBP13 were added to 10 μl of the ubiquitination reaction mixture containing the polyubiquitinated substrates and incubated with deubiquitination buffer (50 mM Tris–HCl [pH 7.4], 150 mM NaCl, 5 mM $MgCl_2$, and 2 mM DTT) at 30°C for 0, 2, and 4 h in a total volume of 30 μl. For co-incubated deubiquitination reactions, three UBP12 derivatives—a partially inactive form of UBP12 in which cysteine 208 was substituted with serine (+UBP12_C208S), the N-terminal region of UBP12 (amino acids 1–541) (+UBP12-N), and the C-terminal region of UBP12 (amino acids 542–1116) (+UBP12-C)—were purified from *E. coli* extracts, as was full-length UBP12 tagged with an N-terminal GST. *In vitro* deubiquitination of MBP-BES1-myc was performed by incubating enzymes E1, E2, and E3 (GST-SINAT2) and His-ubiquitin with full-length UBP12 or its three different derivatives. The deubiquitinating enzymes were added in increasing amounts: 0, 0.1, 0.2, 0.5, and 1 μg. All mixtures were incubated for 4 h at 30°C in a total volume of 30 μl. *E. coli*-derived human tetra K48Ubi (Boston Biochem, cat. no. UC210B) and K63 Ubi (Boston Biochem, cat. no. UC310B) were used as substrates for UBP12/13 and their active-site mutants. Ubiquitinated or deubiquitinated substrates were detected by immunoblotting using anti-myc antibody (Proteintech, cat. no. 16286-1-AP). All assays were repeated in three independent experiments.

Cell-free protein degradation assay

Seven-day-old seedlings grown under normal conditions were frozen with liquid nitrogen and then ground to a powder. After resuspension in a degradation buffer containing 25 mM Tris–HCl (pH 7.4), 10 mM NaCl, 10 mM $MgCl_2$, 5 mM DTT, 10 mM ATP, and protease inhibitor cocktail (Roche, cat. no. 04693159001), the extracts were centrifuged at 12 000 *g* for 10 min at 4°C. Protein concentrations in the supernatants were determined by Bradford assay (Bio-Rad, cat. no. 500-0006), and the supernatants were adjusted to equal protein concentrations. For individual assays, approximately 100 μg of total protein was incubated with 400 ng of the recombinant protein MBP-BES1 in a 100-μl reaction mixture. The reaction mixtures were incubated at the indicated times and temperatures. The collected protein mixtures were separated by SDS–PAGE and analyzed by immunoblotting with anti-MBP antibody (Proteintech, cat. no. 15089-1-AP). Band intensities were measured with ImageJ software. All assays were repeated in three independent experiments.

In vivo ubiquitination assays

In vivo ubiquitination assays were performed using 7-day-old WT (Col-0), *BES1*^{OE}, *BES1*^{OE}/*UBP12*^{OE}, and *BES1*^{OE}/*ubp12-2w/13-3* plants. Seedlings were treated with 1 μM eBL for 6 h. Proteins were extracted with 2× protein extraction buffer as described by Yang et al. (2017) (100 mM Tris–HCl [pH 7.5], 300 mM NaCl, 2 mM EDTA [pH 8.0], 1% Triton X-100, 10% glycerol, 50 μM MG132, and protease inhibitor), and extracts were centrifuged at 12 000 rpm for 10 min at 4°C. After pre-clearing for 1 h, total soluble protein extracts were incubated with TUBE magnetic beads (LifeSensors, cat. no. UM-0402M) at 4°C for 4 h. The magnetic beads were washed 4 times with 1× protein extraction buffer. The pooled protein mixtures were separated by SDS–PAGE and analyzed by immunoblotting with anti-myc antibody (Proteintech, cat. no. 16286-1-AP) and anti-Ubi antibody (Santa Cruz Biotechnology, P4D1, cat. no. sc-8017). All assays were repeated in three independent experiments.

ACCESSION NUMBERS

Genes and the accession numbers used in this study are as follows: *UBP12* (At5g06600), *UBP13* (At3g11910), *BES1* (At1g19350), *BZR1* (At1g75080), *SINAT2* (At3g58040), *CPD* (At5g05690), *DWF4* (At3g50660), *ACS5* (At5g65800), *SAUR-AC1* (At4g38850), and *ACT2* (At3g18780). Sequence information was extracted from The Arabidopsis Information Resource (http://www.arabidopsis.org/).

SUPPLEMENTAL INFORMATION

Supplemental information is available at *Plant Communications Online*.

FUNDING

This work was supported in part by an RSSS grant (no. NRF-RSSS-002) to N.-H.C. from the National Research Foundation, Singapore.

AUTHOR CONTRIBUTIONS

S.-H.P. and N.-H.C. designed the experiments. S.-H.P., J.S.J., Y.Z., and N.F.B.M. performed the experiments. S.-H.P. and N.-H.C. analyzed the data and wrote the manuscript.

ACKNOWLEDGMENTS

No conflict of interest declared.

Received: August 24, 2021
Revised: June 6, 2022
Accepted: June 9, 2022
Published: June 15, 2022

REFERENCES

An, Z., Liu, Y., Ou, Y., Li, J., Zhang, B., Sun, D., Sun, Y., and Tang, W. (2018). Regulation of the stability of RGF1 receptor by the ubiquitin-specific proteases UBP12/UBP13 is critical for root meristem maintenance. Proc. Natl. Acad. Sci. USA **115**:1123–1128. https://doi.org/10.1073/pnas.1714177115.

Callis, J. (2014). The ubiquitination machinery of the ubiquitin system. Arabidopsis Book **12**:e0174. https://doi.org/10.1199/tab.0174.

Clough, S.J., and Bent, A.F. (1998). Floral dip: a simplified method for Agrobacterium-mediated transformation of Arabidopsis thaliana. Plant J. **16**:735–743. https://doi.org/10.1046/j.1365-313x.1998.00343.x.

Cui, X., Lu, F., Li, Y., Xue, Y., Kang, Y., Zhang, S., Qiu, Q., Cui, X., Zheng, S., Liu, B., et al. (2013). Ubiquitin-specific proteases UBP12 and UBP13 act in circadian clock and photoperiodic flowering regulation in *Arabidopsis*. Plant Physiol. **162**:897–906. https://doi.org/10.1104/pp.112.213009.

Derkacheva, M., Liu, S., Figueiredo, D.D., Gentry, M., Mozgova, I., Nanni, P., Tang, M., Mannervik, M., Köhler, C., and Hennig, L. (2016). H2A deubiquitinases UBP12/13 are part of the Arabidopsis polycomb group protein system. Nature Plants **2**:16126. https://doi.org/10.1038/nplants.2016.126.

Dong, H., Dumenil, J., Lu, F.H., Na, L., Vanhaeren, H., Naumann, C., Klecker, M., Prior, R., Smith, C., McKenzie, N., et al. (2017). Ubiquitylation activates a peptidase that promotes cleavage and destabilization of its activating E3 ligases and diverse growth regulatory proteins to limit cell proliferation in Arabidopsis. Genes Dev. **31**:197–208. https://doi.org/10.1101/gad.292235.116.

Eletr, Z.M., and Wilkinson, K.D. (2014). Regulation of proteolysis by human deubiquitinating enzymes. Biochim. Biophys. Acta **1843**:114–128. https://doi.org/10.1016/j.bbamcr.2013.06.027.

Erpapazoglou, Z., Walker, O., and Haguenauer-Tsapis, R. (2014). Versatile roles of K63-linked ubiquitin chains in trafficking. Cells **3**:1027–1088. https://doi.org/10.3390/cells3041027.

Ewan, R., Pangestuti, R., Thornber, S., Craig, A., Carr, C., O'Donnell, L., Zhang, C., and Sadanandom, A. (2011). Deubiquitinating enzymes AtUBP12 and AtUBP13 and their tobacco homologue NtUBP12 are negative regulators of plant immunity. New Phytol. **191**:92–106. https://doi.org/10.1111/j.1469-8137.2011.03672.x.

Gampala, S.S., Kim, T.W., He, J.X., Tang, W., Deng, Z., Bai, M.Y., Guan, S., Lalonde, S., Sun, Y., Gendron, J.M., et al. (2007). An essential role for 14-3-3 proteins in brassinosteroid signal transduction in Arabidopsis. Dev. Cell **13**:177–189. https://doi.org/10.1016/j.devcel.2007.06.009.

Griffiths, J., Murase, K., Rieu, I., Zentella, R., Zhang, Z.L., Powers, S.J., Gong, F., Phillips, A.L., Hedden, P., Sun, T.P., et al. (2006). Genetic characterization and functional analysis of the GID1 gibberellin receptors in Arabidopsis. Plant Cell **18**:3399–3414. https://doi.org/10.1105/tpc.106.047415.

House, C.M., Frew, I.J., Huang, H.L., Wiche, G., Traficante, N., Nice, E., Catimel, B., and Bowtell, D.D.L. (2003). A binding motif for Siah ubiquitin ligase. Proceedings of the National Academy of Sciences, USA **100**:3101–3106. https://doi.org/10.1073/pnas.0534783100.

Jeong, J.S., Jung, C., Seo, J.S., Kim, J.-K., and Chua, N.-H. (2017). The deubiquitinating enzymes UBP12 and UBP13 positively regulate MYC2 levels in Jasmonate responses. Plant Cell **29**:1406–1424. https://doi.org/10.1105/tpc.17.00216.

Isono, E., and Nagel, M.-K. (2014). Deubiquitylating enzymes and their emerging role in plant biology. Front. Plant Sci. **5**:56. https://doi.org/10.3389/fpls.2014.00056.

Kasahara, H. (2016). Current aspects of auxin biosynthesis in plants. Biosci., Biotechnol., Biochem. **80**:34–42. https://doi.org/10.1080/09168451.2015.1086259.

Kim, B., Jeong, Y.J., Corvalán, C., Fujioka, S., Cho, S., Park, T., and Choe, S. (2014). Darkness and gulliver2/phyB mutation decrease the abundance of phosphorylated BZR1 to activate brassinosteroid signaling in *Arabidopsis*. Plant J. **77**:737–747. https://doi.org/10.1111/tpj.12423.

Kim, R.Q., van Dijk, W.J., and Sixma, T.K. (2016). Structure of USP7 catalytic domain and three Ubl-domains reveals a connector α-helix with regulatory role. J. Struct. Biol. **195**:11–18. https://doi.org/10.1016/j.jsb.2016.05.005.

Kim, E.-J., Lee, S.-H., Park, C.-H., Kim, S.-H., Hsu, C.-C., Xu, S., Wang, Z.-Y., Kim, S.-K., and Kim, T.-W. (2019). Plant U-Box40 mediates degradation of the brassinosteroid-responsive transcription factor BZR1 in *Arabidopsis* roots. Plant Cell **31**:791–808. https://doi.org/10.1105/tpc.18.00941.

Koornneef, M., and van der Veen, J.H. (1980). Induction and analysis of gibberellin-sensitive mutants in Arabidopsis thaliana (L.) Heynh. Theor. Appl. Genet. **58**:257–263. https://doi.org/10.1007/bf00265176.

Lee, C.-M., Li, M.-W., Feke, A., Liu, W., Saffer, A.M., and Gendron, J.M. (2019). GIGANTEA recruits the UBP12 and UBP13 deubiquitylases to regulate accumulation of the ZTL photoreceptor complex. Nat. Commun. **10**:3750. https://doi.org/10.1038/s41467-019-11769-7.

Li, J., Nam, K.H., Vafeados, D., and Chory, J. (2001). BIN2, a new brassinosteroid-insensitive locus in Arabidopsis. Plant Physiol. **127**:14–22. https://doi.org/10.1104/pp.127.1.14.

Li, J., and Nam, K.H. (2002). Regulation of brassinosteroid signaling by a GSK3/SHAGGY-like kinase. Science **295**:1299–1301. https://doi.org/10.1126/science.1065769.

Li, Q.-F., Lu, J., Yu, J.-W., Zhang, C.Q., He, J.-X., and Liu, Q.-Q. (2018). The brassinosteroid-regulated transcription factors BZR1/BES1 function as a coordinator in multisignal-regulated plant growth. BBA - Gene Regulatory Mechanisms **1861**:561–571. https://doi.org/10.1016/j.bbagrm.2018.04.003.

Luo, Y., Takagi, J., Alves Neubus Claus, L., Zhang, C., Yasuda, S., Hasegawa, Y., Yamaguchi, J., Shan, L., Russinova, E., and Sato,

T. (2022). Deubiquitinating enzymes UBP12 and UBP13 stabilize the brassinosteroid receptor BRI1. EMBO Rep. e53354.

Lv, M., and Li, J. (2020). Molecular mechanisms of brassinosteroid-mediated responses to changing environments in Arabidopsis. Int. J. Mol. Sci. **21**:2737. https://doi.org/10.3390/ijms21082737.

March, E., and Farrona, S. (2018). Plant deubiquitinases and their role in the control of gene expression through modification of histones. Front. Plant Sci. **8**:2274. https://doi.org/10.3389/fpls.2017.02274.

Marshall, R.S., Li, F., Gemperline, D.C., Book, A.J., and Vierstra, R.D. (2015). Autophagic degradation of the 26S proteasome is mediated by the dual ATG8/ubiquitin receptor RPN10 in Arabidopsis. Mol. Cell **58**:1053–1066. https://doi.org/10.1016/j.molcel.2015.04.023.

Majumdar, P., and Nath, U. (2020). De-ubiquitinases on the move: an emerging field in plant biology. Plant Biol. **22**:563–572. https://doi.org/10.1111/plb.13118.

Nolan, T.M., Brennan, B., Yang, M., Chen, J., Zhang, M., Li, Z., Wang, X., Bassham, D.C., Walley, J., and Yin, Y. (2017). Selective autophagy of BES1 mediated by DSK2 balances plant growth and survival. Dev. Cell **41**:33–46.e7. https://doi.org/10.1016/j.devcel.2017.03.013.

Nolan, T.M., Vukašinović, N., Liu, D., Russinova, E., and Yin, Y. (2020). Brassinosteroids: multidimensional regulators of plant growth, development, and stress responses. Plant Cell **32**:295–318. https://doi.org/10.1105/tpc.19.00335.

Oh, M.-H., Honey, S.H., and Tax, F.E. (2020). The control of cell expansion, cell division, and vascular development by brassinosteroids: a historical perspective. Int. J. Mol. Sci. **21**:1743. https://doi.org/10.3390/ijms21051743.

Ohri, P., Bhardwaj, R., Kaur, R., Jasrotia, S., Parihar, R.D., Khajuria, A., and Sharma, N. (2019). Emerging trends on crosstalk of Brs with other phytohormones. *Brassinosteroids*. Plant Growth Dev. 425–441.

Park, S.-H., Jeong, J.S., Seo, J.S., Park, B.S., and Chua, N.-H. (2019). Arabidopsis ubiquitin-specific proteases UBP12 and UBP13 shape ORE1 levels during leaf senescence induced by nitrogen deficiency. New Phytol. **223**:1447–1460. https://doi.org/10.1111/nph.15879.

Romero-Barrios, N., and Vert, G. (2018). Proteasome-independent functions of lysine-63 polyubiquitination in plants. New Phytol. **217**:995–1011. https://doi.org/10.1111/nph.14915.

Ryu, H., Kim, K., Cho, H., Park, J., Choe, S., and Hwang, I. (2007). Nucleocytoplasmic shuttling of BZR1 mediated by phosphorylation is essential in Arabidopsis brassinosteroid signaling. Plant Cell **19**:2749–2762. https://doi.org/10.1105/tpc.107.053728.

Sun, Y., Fan, X.Y., Cao, D.M., Tang, W., He, K., Zhu, J.Y., He, J.X., Bai, M.Y., Zhu, S., Oh, E., et al. (2010). Integration of brassinosteroid signal transduction with the transcription network for plant growth regulation in *Arabidopsis*. Dev. Cell **19**:765–777. https://doi.org/10.1016/j.devcel.2010.10.010.

Vanhaeren, H., Chen, Y., Vermeersch, M., De Milde, L., De Vleeschhauwer, V., Natran, A., Persiau, G., Eeckhout, D., De Jaeger, G., Gevaert, K., et al. (2020). UBP12 and UBP13 negatively regulate the activity of the ubiquitin-dependent peptidases DA1, DAR1 and DAR2. Elife **9**:e52276. https://doi.org/10.7554/elife.52276.

Vierstra, R.D. (2009). The ubiquitin-26S proteasome system at the nexus of plant biology. Nat. Rev. Mol. Cell Biol. **10**:385–397. https://doi.org/10.1038/nrm2688.

Wang, F., and Deng, X.W. (2011). Plant ubiquitin-proteasome pathway and its role in gibberellin signaling. Cell Res. **21**:1286–1294. https://doi.org/10.1038/cr.2011.118.

Wang, C., Shang, J.X., Chen, Q.X., Oses-Prieto, J.A., Bai, M.Y., Yang, Y., Yuan, M., Zhang, Y.L., Mu, C.C., Deng, Z., et al. (2013a). Identification of BZR1-interacting proteins as potential components of the brassinosteroid signaling pathway in Arabidopsis through tandem affinity purification. Mol. Cell. Proteomics **12**:3653–3665. https://doi.org/10.1074/mcp.m113.029256.

Wang, P., Nolan, T.M., Clark, N.M., Jiang, H., Montes-Serey, C., Guo, H., Bassham, D.C., Walley, J.W., and Yin, Y. (2021a). The F-box E3 ubiquitin ligase BAF1 mediates the degradation of the brassinosteroid-activated transcription factor BES1 through selective autophagy in Arabidopsis. Plant Cell **33**:3532–3554. https://doi.org/10.1093/plcell/koab210.

Wang, R., Wang, R., Liu, M., Yuan, W., Zhao, Z., Liu, X., Peng, Y., Yang, X., Sun, Y., and Tang, W. (2021b). Nucleocytoplasmic trafficking and turnover mechanisms of BRASSINAZOLE RESISTANT1 in Arabidopsis thaliana. Proc. Natl. Acad. Sci. USA **118**, e2101838118. https://doi.org/10.1073/pnas.2101838118.

Wang, Y., Sun, S., Zhu, W., Jia, K., Yang, H., and Wang, X. (2013b). Strigolactone/MAX2-induced degradation of brassinosteroid transcriptional effector BES1 regulates shoot branching. Dev. Cell **27**:681–688. https://doi.org/10.1016/j.devcel.2013.11.010.

Yan, N., Doelling, J.H., Falbel, T.G., Durski, A.M., and Vierstra, R.D. (2000). The ubiquitin-specific protease family from Arabidopsis, AtUBP1 and 2 are required for the resistance to the amino acid analog canavanine. Plant Physiol. **124**:1828–1843. https://doi.org/10.1104/pp.124.4.1828.

Yang, M., Li, C., Cai, Z., Hu, Y., Nolan, T., Yu, F., Yin, Y., Xie, Q., Tang, G., and Wang, X. (2017). SINAT E3 ligases control the light-mediated stability of the brassinosteroid-activated transcription factor BES1 in *Arabidopsis*. Dev. Cell **41**:47–58.e4. https://doi.org/10.1016/j.devcel.2017.03.014.

Yin, Y., Vafeados, D., Tao, Y., Yoshida, S., Asami, T., and Chory, J. (2005). A new class of transcription factors mediates brassinosteroid-regulated gene expression in Arabidopsis. Cell **120**:249–259. https://doi.org/10.1016/j.cell.2004.11.044.

Yu, X., Li, L., Zola, J., Aluru, M., Ye, H., Foudree, A., Guo, H., Anderson, S., Aluru, S., Liu, P., et al. (2011). A brassinosteroid transcriptional network revealed by genome-wide identification of BES1 target genes in *Arabidopsis thaliana*. Plant J. **65**:634–646. https://doi.org/10.1111/j.1365-313x.2010.04449.x.

Zhang, X., Henriques, R., Lin, S.S., Niu, Q.W., and Chua, N.-H. (2006). Agrobacterium-mediated transformation of Arabidopsis thaliana using the floral dip method. Nat. Protoc. **1**:641–646. https://doi.org/10.1038/nprot.2006.97.

Zhou, Y., Park, S.-H., Soh, M.Y., and Chua, N.-H. (2021). Ubiquitin-specific proteases UBP12 and UBP13 promote shade avoidance response by enhancing PIF7 stability. Proc. Natl. Acad. Sci. USA **118**, e2103633118. https://doi.org/10.1073/pnas.2103633118.

Zientara-Rytter, K., and Subramani, S. (2019). The roles of ubiquitin-binding protein shuttles in the degradative fate of ubiquitinated proteins in the ubiquitin-proteasome system and autophagy. Cells **8**:40. https://doi.org/10.3390/cells8010040.

Plant Communications, Volume *3*

Supplemental information

Deubiquitination of BES1 by UBP12/UBP13 promotes brassinosteroid signaling and plant growth

Su-Hyun Park, Jin Seo Jeong, Yu Zhou, Nur Fatimah Binte Mustafa, and Nam-Hai Chua

Supplemental information

Deubiquitination of BES1 by UBP12/UBP13 promotes brassinosteroid signaling and plant growth

Su-Hyun Park[1], Jin Seo Jeong[1,2], Yu Zhou[1], Nur Fatimah Binte Mustafa[1], Nam-Hai Chua[1,*]

[1]Temasek Life Sciences Laboratory, 1 Research Link, National University of Singapore, Singapore 117604, Singapore

[2] Present address: Wilmar International Limited, 28 Biopolis Road, Singapore 138568, Singapore

*Corresponding author: Nam-Hai Chua; chua@rockefeller.edu

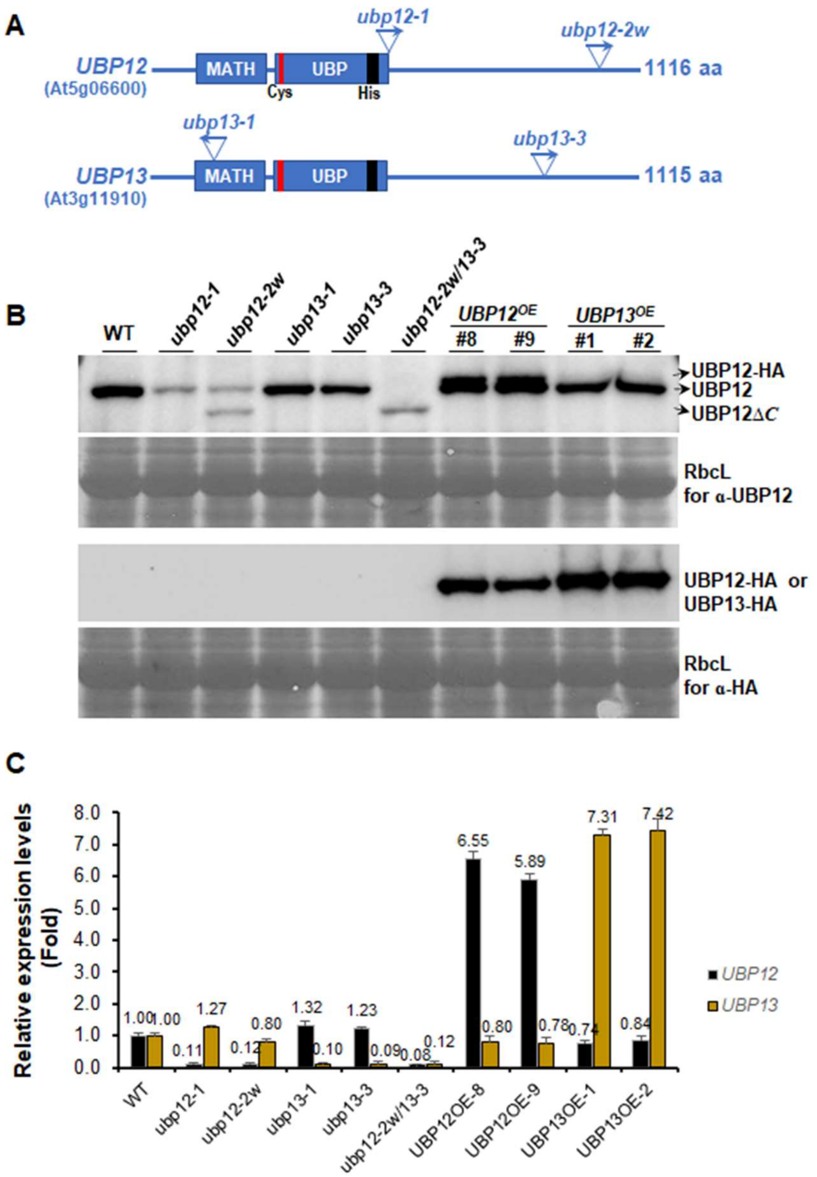

Figure S1. Molecular characterization of plants deficient in UBP12/UBP13 and plants overexpressing these proteins.

(A) Schematic gene structures of *UBP12* and *UBP13* with T-DNA insertions and encoded protein domains. MATH, meprin and tumor necrosis factor receptor-associated factor homology domain; UBP, Ubiquitin-specific protease domain containing Cys and His motifs. **(B)** UBP12/UBP13 protein levels in 4 single mutants, *ubp12-1*, *ubp12-2w*, *ubp13-1*, *ubp13-3*, one double mutant, *ubp12-2w/13-3,* and 2 independent transgenic lines over-expressing *UBP12* (*UBP12^{OE}*) or *UBP13* (*UBP13^{OE}*). Seven-day-old seedlings grown under normal conditions were used. Endogenous UBP12 and UBP13 were detected by immunoblots using anti-UBP12

antibody, which reacted with both homologous proteins. Transgenic UBP12-HA and UBP13-HA in *UBP12*^{OE} and *UBP13*^{OE} plants were detected using an anti-HA antibody. UBP12Δ*C* indicates a truncated form of UBP12 due to the T-DNA insertion in *ubp12-2w*. RbcL protein levels were used as a loading control. Western blot assays were analyzed in three independent experiments and images from one representative set were shown. **(C)** Relative gene expression levels of *UBP12* and *UBP13* in various plants. Transcript levels of *UBP12* and *UBP13* were analyzed in 7-d-old seedlings by qRT-PCR analysis. Expression levels of WT were set as 1. All qRT-PCR experiments were performed in triplicates. Average values of 3 independent biological samples (*n*=3) were presented with standard deviation. *ACT2* expression levels were used as a control for normalization.

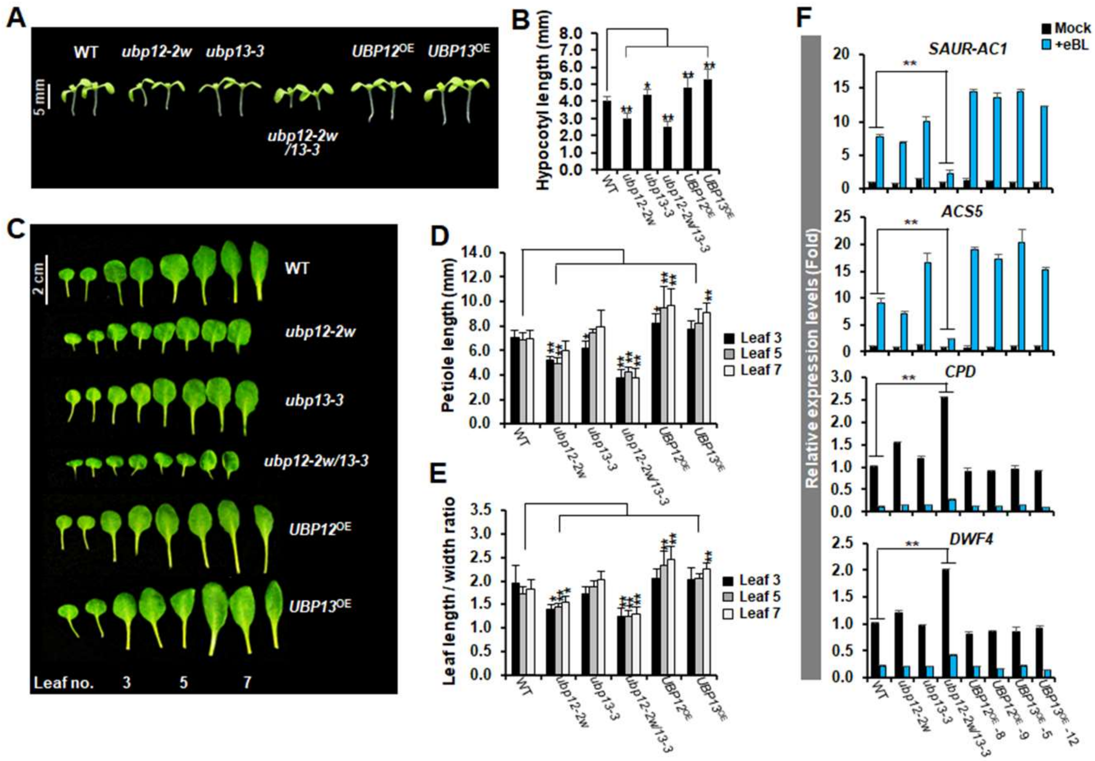

Figure S2. BR-deficient phenotypes of *ubp12-2w/13-3* plants.

(A) Phenotypes of WT (Col-0), *ubp12-2w*, *ubp13-3*, *ubp12-2w/13-3,* and *UBP12*- or *UBP13*-overexpressing plants (*UBP12^{OE}* or *UBP13^{OE}*). Seedlings were grown on ½ MS medium under dim light (25 µmol $m^{-2}s^{-1}$) for 7 days. Bar, 5 mm. **(B)** Hypocotyl lengths of seedlings indicated in **(A)**. **(C-E)** Phenotypes of rosette leaves of various genotypes grown on ½ MS medium for 3 weeks under normal light (100 µmol $m^{-2}s^{-1}$). Bar, 2 cm. The petiole length (mm) and the ratio of leaf length to width of the 3rd, 5th, and 7th leaves are shown in **(D)** and **(E)**. Fifteen independent biological samples (*n*=15) were analyzed and one image is shown as a representative. *, $P < 0.05$; **, $P < 0.01$ (two-tailed *t-test*). **(F)** Changes in BR-responsive gene expression in plants deficient in or overexpressing UBP12/UBP13. Transcript levels of genes repressed- (*CPD* and *DWF4*) or induced- (*SAUR-AC1* and *ACS5*) by brassinosteroids (BR) were analyzed in 7-d-old seedlings treated with 1 µM of epi-brassinolide (eBL) for 6 hours by qRT-PCR analysis. Expression levels of WT were set as 1. All qRT-PCR experiments were performed in triplicates. Average values of 3 independent biological samples (*n*=3) were presented with standard deviation. *ACT2* expression levels were used as a control for normalization.

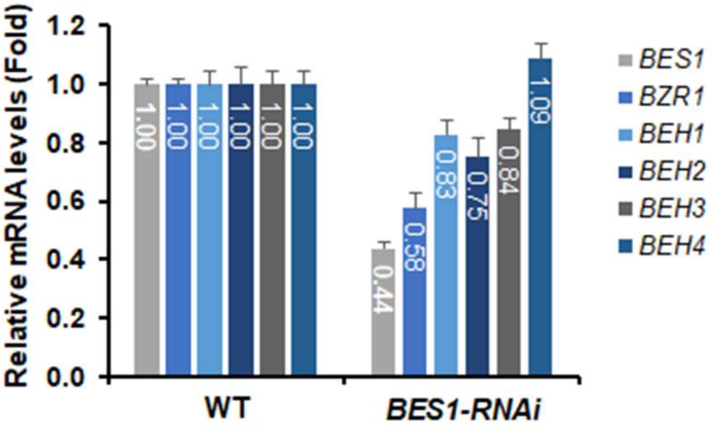

Figure S3. Expression of *BES1* family genes in *BES1-RNAi* lines.

Transcript levels of *BES1* family genes were analysed by qRT-PCR using 7-day-old seedlings grown in normal conditions. All qRT-PCR experiments were performed in triplicates and the average values were presented with S.D. (standard deviation) (n=3). *ACT2* expression levels were used as a control for normalization.

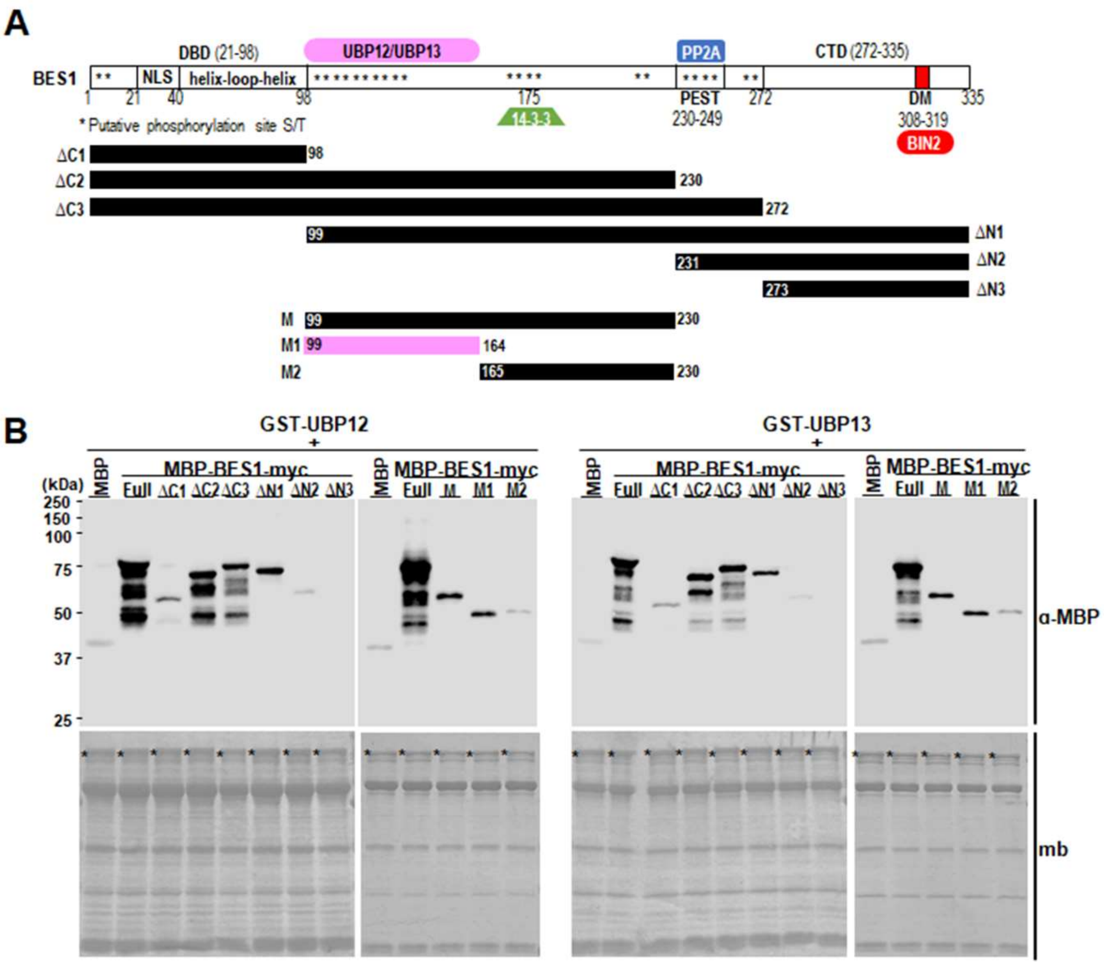

Figure S4. Interaction of UBP12/UBP13 with BES1 fragments.

(A) Schematic diagram of various truncated forms of BES1 (upper panel). Numbers indicate the amino acid positions of the derivatives. **(B)** Truncated forms of BES1 like ΔC1, ΔC2, ΔC3, ΔN1, ΔN2, and ΔN3, and the middle part of BES1 (M) were dissected into two regions like M1 and M2, which were pulled down by GST-UBP12 and GST-UBP13. 'Full' denotes the full-length BES1. MBP was used as a negative control. Two µg of each protein was used in all experiments. Protein bands were detected by immunoblots using anti-MBP antibody and the membrane (mb) was stained with Coomassie Brilliant Blue. The asterisk marks resin-bound bait proteins like GST-UBP12 and GST-UBP13. Three independent experiments were performed and images from one representative set are shown.

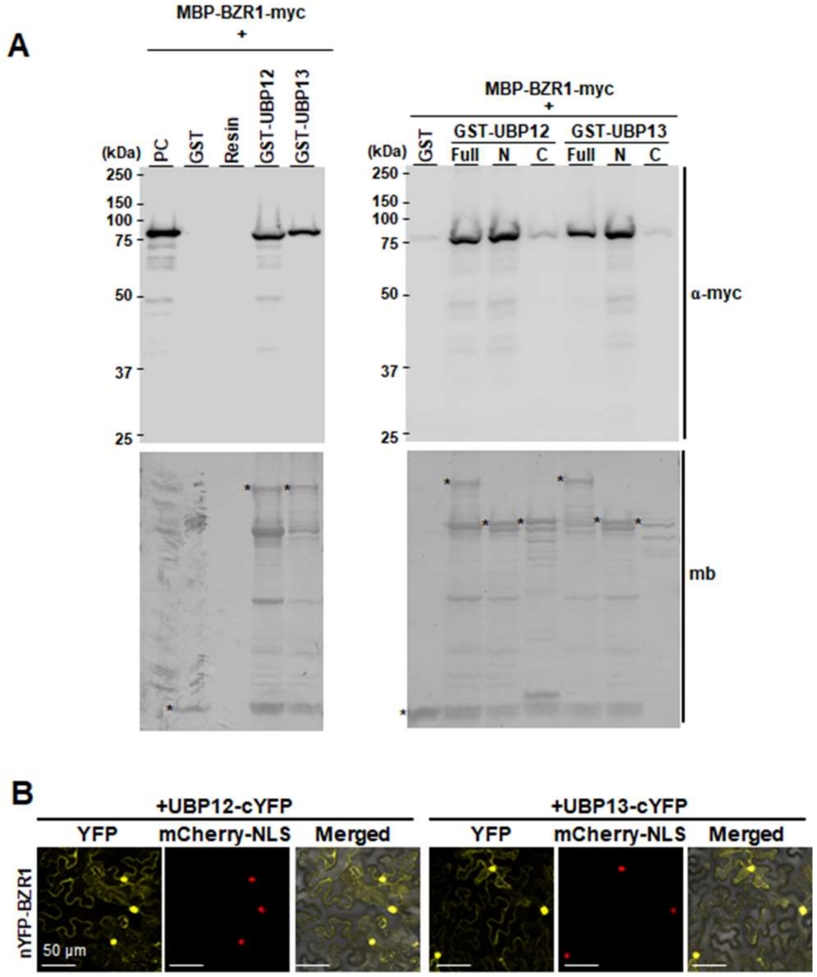

Figure S5. UBP12/UBP13 and BZR1 interaction *in vitro*.

(A) MBP-BZR1-myc was pulled down by GST resin-bound full-length UBP12/UBP13 (Full) and derivatives carrying the N- or C-terminal region (N or C) as shown in Figure 3A. Bands were detected by immunoblots using anti-myc antibody. Membrane (mb) was stained with Coomassie Brilliant Blue. Two µg of each protein was used in all experiments. The positive control (PC) sample contained 50 ng MBP-BZR1-myc. The asterisk marks resin-bound bait proteins. MBP, maltose-binding protein; GST, glutathione-S-transferase, kDa, kilodalton. **(B)** Interaction between UBP12/UBP13 and BZR1 in tobacco (*Nicotiana benthamiana*) leaves assayed by Bimolecular Fluorescence Complementation (BiFC). nYFP-BZR1 was transiently co-expressed with UBP12-cYFP or UBP13-cYFP in leaves of *N. benthamiana* infiltrated with *Agrobacterium*. Localization is shown by merging YFP, mCherry-NLS (Nuclear Localization Sequence) and DIC images (Merged). Confocal microscopic images were taken 3 days after infiltration. DIC, differential interference contrast, Bars, 50 µm. Western blots and BiFC assays were analyzed in 3 independent experiments and images of a representative set are shown.

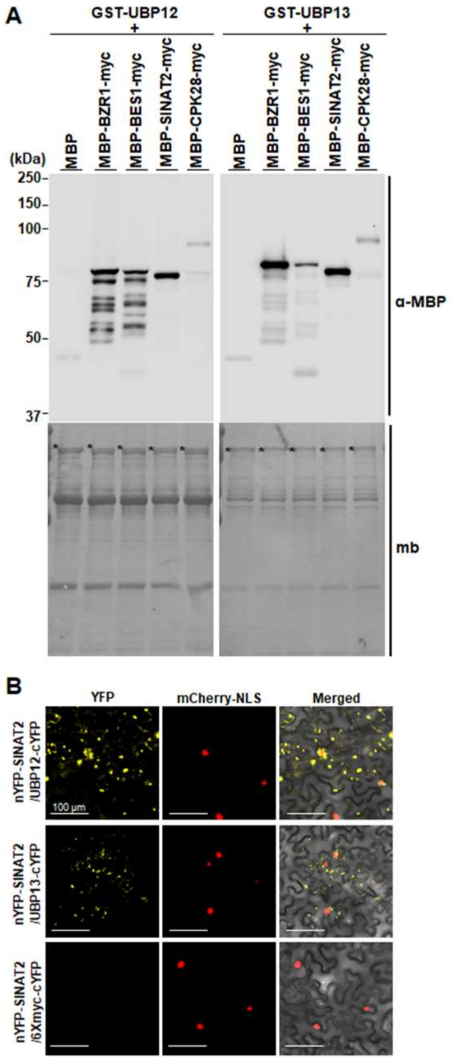

Figure S6. In vitro and in vivo interaction between UBP12/UBP13 and SINAT2.

(A) UBP12/UBP13 and SINAT2 interaction *in vitro*. MBP-SINAT2-myc was pulled down by UBP12/UBP13 tagged with GST. Protein bands were detected by immunoblots using anti-myc antibody. Membrane (mb) was stained with Coomassie Brilliant Blue. MBP and MBP-CPK28-myc were used as negative controls. MBP-BZR1-myc and MBP-BES1-myc were used as positive controls. Two µg of each protein was used in all experiments. The asterisk marks resin-bound bait proteins. MBP, maltose-binding protein; GST, glutathione-S-transferase, kDa, kilodalton. **(B)** In vivo interaction between UBP12/UBP13 and SINAT2 assayed by Bimolecular Fluorescence Complementation (BiFC). *SINAT2* fused with *nYFP* was transiently co-expressed with *UBP12-cYFP* and/or *UBP13-cYFP* in tobacco leaves (*N. benthamiana*) infiltrated with *Agrobacterium*. Localization is shown by merging YFP, mCherry-NLS (Nuclear Localization Sequence), and DIC images (Merged). Confocal microscopic images were taken 3 days after infiltration. DIC, differential interference contrast. Bars, 100 µm.

Western blots and BiFC assays were analyzed in 3 independent experiments and images of a representative set are shown.

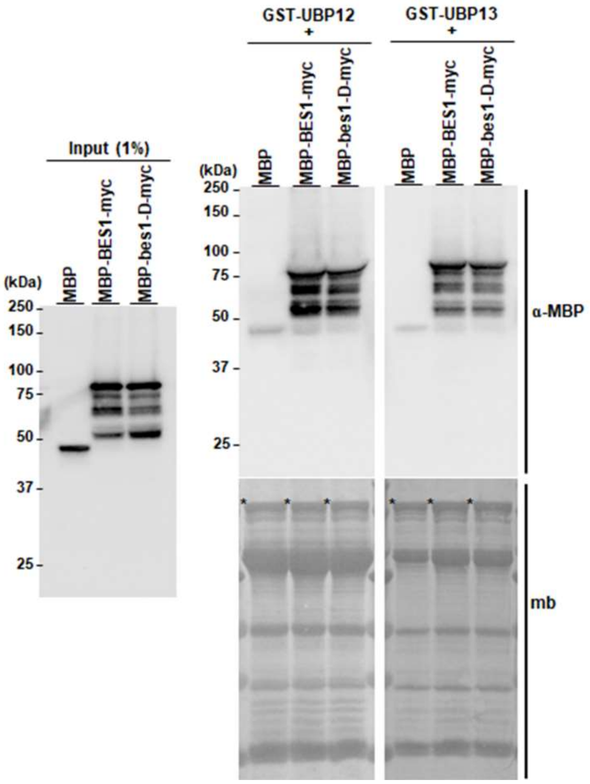

Figure S7. In vitro interaction between UBP12/UBP13 and bes1-D.

(A) UBP12/UBP13 and bes1-D interaction *in vitro*. MBP-bes1-D-myc was pulled down by UBP12/UBP13 tagged with GST. Protein bands were detected by immunoblots using anti-myc antibody. Membrane (mb) for GST-UBP12 and GST-UBP13 was stained with Coomassie Brilliant Blue. MBP was used as a negative control and MBP-BES1-myc was used as a positive control. Two µg of each protein was used in all experiments. The asterisk marks resin-bound bait proteins. MBP, maltose-binding protein; GST, glutathione-S-transferase, kDa, kilodalton.

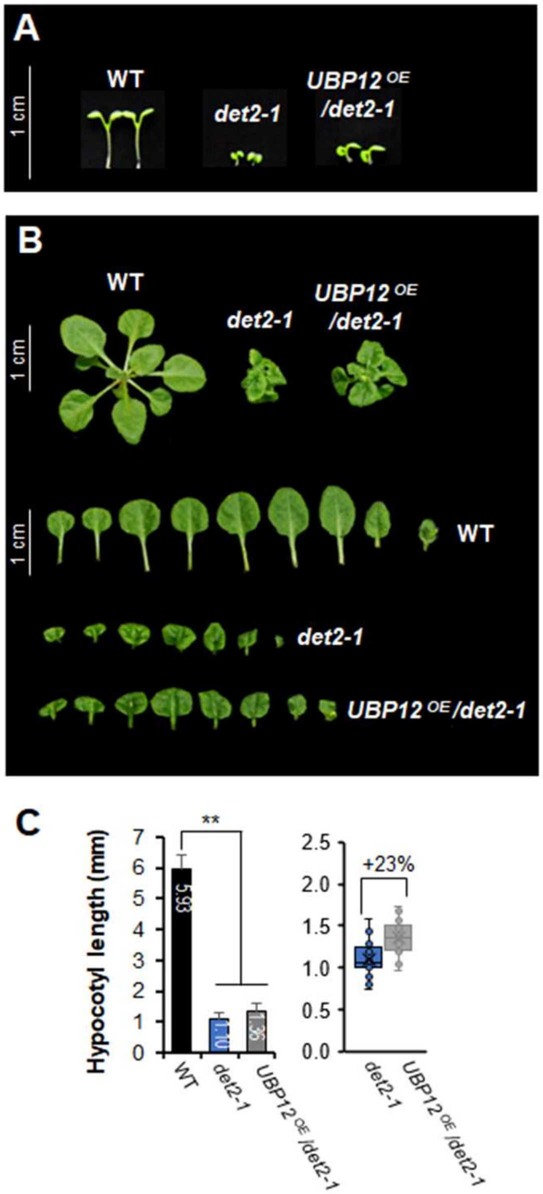

Figure S8. Partial restoration of *det2-1* BR-deficient phenotypes by *UBP12* overexpression.

(A) Seedlings for WT, *det2-1*, and *UBP12^{OE}/det2-1* were grown on ½ MS medium under dim light (25 µmol $m^{-2}s^{-1}$) for 7 days. The *UBQ10:UBP12-HA* construct was used for over-expression. Bar, 1 cm. **(B)** Phenotypes of rosette leaves of various genotypes grown on ½ MS medium for 3 weeks under normal light (100 µmol $m^{-2}s^{-1}$). Bar, 1 cm. **(C)** Hypocotyl lengths of seedlings indicated in **(A)**. Fifteen independent biological samples (n=15) were analyzed and one image is shown as a representative. *, $P < 0.05$; **, $P < 0.01$ (two-tailed *t-test*).

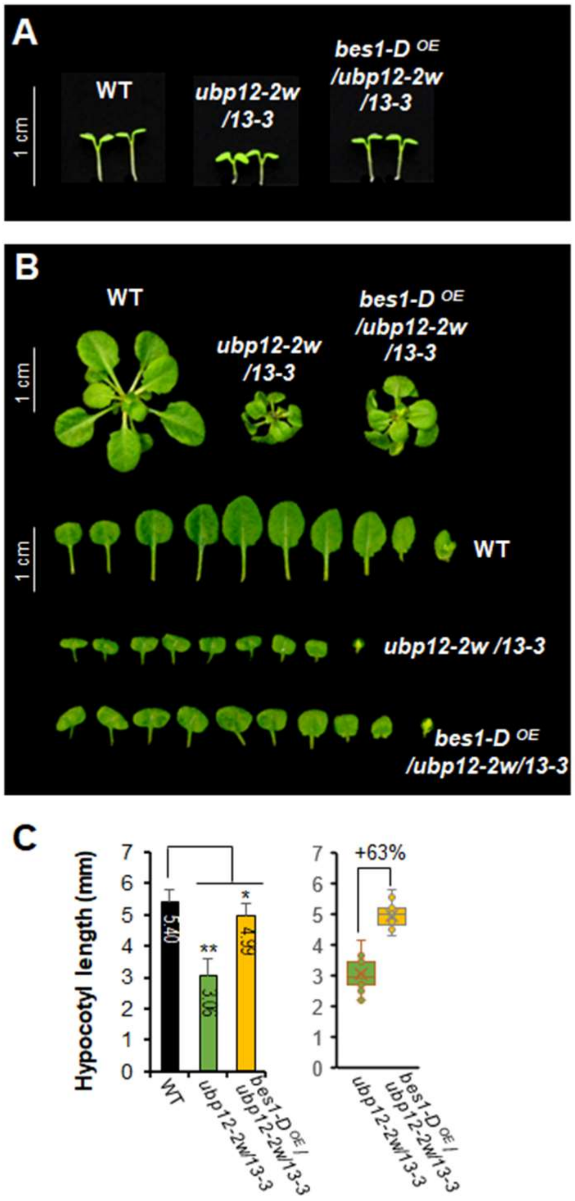

Figure S9. Partial restoration of *ubp12-2w/13-3* BR-deficient phenotypes by *bes1-D* overexpression.

(A) Seedlings for WT, *ubp12-2w/13-3* and *bes1-D*^{OE}*/ubp12-2w/13-3* were grown on ½ MS medium under dim light (25 µmol $m^{-2}s^{-1}$) for 7 days. *35S:bes1-D-YFP* construct was used for over-expression. Bar, 1 cm. **(B)** Phenotypes of rosette leaves of various genotypes grown on ½ MS medium for 3 weeks under normal light (100 µmol $m^{-2}s^{-1}$). Bar, 1 cm. **(C)** Hypocotyl lengths of seedlings indicated in **(A)**. Fifteen independent biological samples (*n*=15) were analyzed and one image is shown as a representative. *, *P* < 0.05; **, *P* < 0.01 (two-tailed *t-test*).

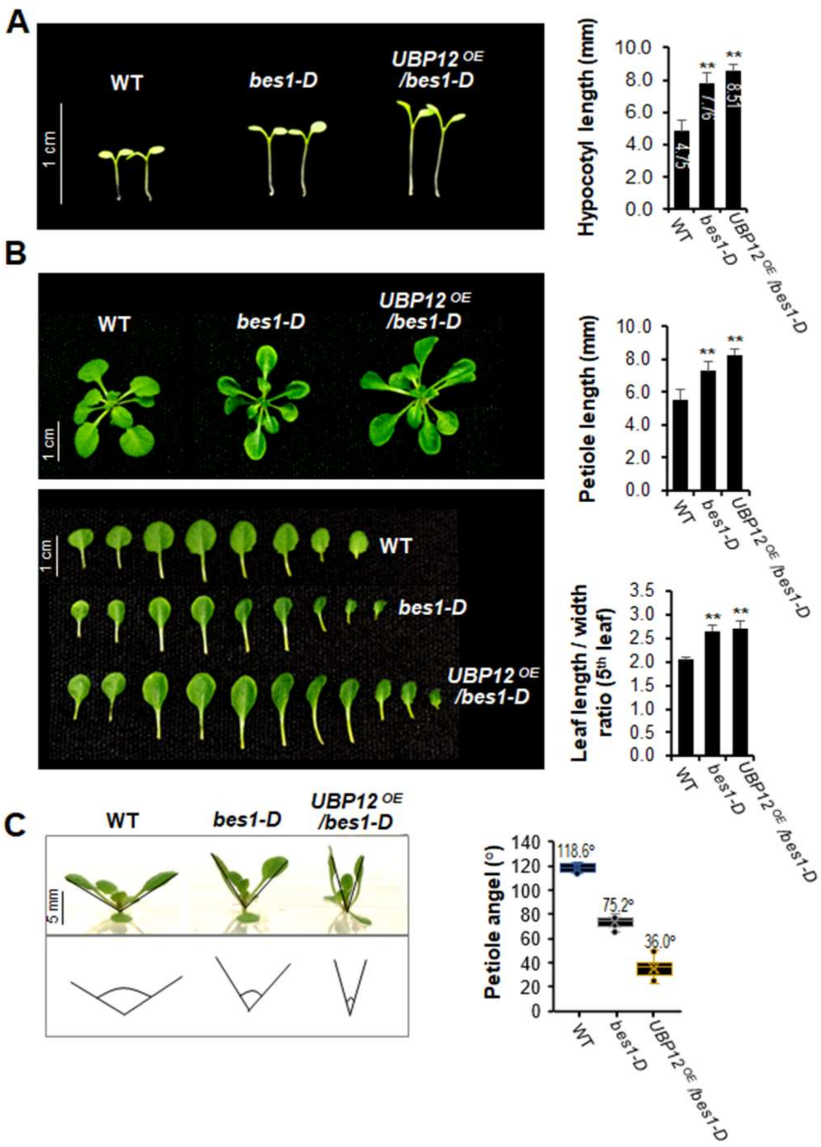

Figure S10. *UBP12* overexpression promotes growth of *bes1-D* plants.

“(A) Seedlings of WT, *bes1-D* (CS65988) and *UBP12*^{OE}/*bes1-D* were grown on ½ MS medium under dim light (25 µmol m^{-2}s^{-1}) for 7 days. The *UBQ10:UBP12-HA* construct was used for over-expression. Bar, 1 cm. Hypocotyl lengths (mm) of seedlings are indicated on the right panel. **(B)** Phenotypes of rosette leaves of various genotypes grown on ½ MS medium for 3 weeks under normal light (100 µmol m^{-2}s^{-1}). Bar, 1 cm. The petiole length (mm) and the ratio of leaf length to width of the 5th leaves are shown on the right panels. **(C)** Petiole angles of 2-week-old plants. Fifteen independent biological samples (*n*=15) were analyzed and one representative image is shown. *, *P* < 0.05; **, *P* < 0.01 (two-tailed *t-test*).

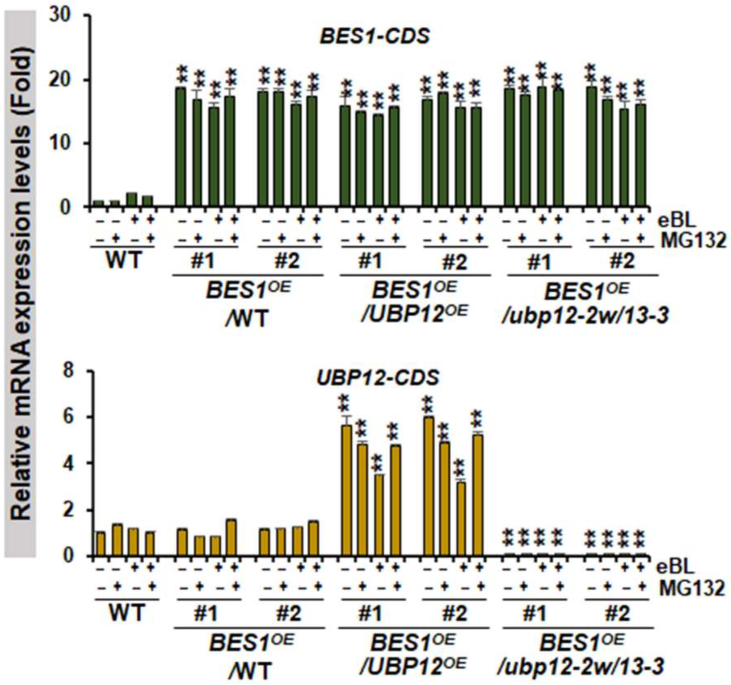

Figure S11. ***BES1*** **and** ***UBP12*** **transcript levels of** ***BES1^{OE}*****/WT,** ***BES1^{OE}/UBP12^{OE}*****, and** ***BES1^{OE}/ubp12-2w/13-3*****.**

Quantitative RT-PCR analysis of *BES1* and *UBP12* transcript levels of *BES1^{OE}*/WT, *BES1^{OE}/UBP12^{OE}*, and *BES1^{OE}/ubp12-2w/13-3* treated with/without MG132 or eBL as shown in figure 5F. Samples prepared from whole seedlings were used for immunoblotting and qRT-PCR analysis. Transcript levels of *BES1* and *UBP12* were examined by qRT-PCR with primers specific for each coding sequence (CDS). All qRT-PCR experiments were performed in triplicates. Expression values of WT were set as 1. Average values from 3 independent biological experiments (n=3) were shown with standard deviation, SD (±). *ACT2* expression levels were used as a control for normalization. *, $P < 0.05$; **, $P < 0.01$ (two-tailed *t-test*).

Table S1 Primer list used in this study

Gene	Purpose	Primer Sequence: Forward		Primer Sequence: Reverse	
UBP12	CDS	5'- ATGACTATGATGACTCCGCCTCCCGTTGAT	-3'	5'- ATTGTATATTTTTACCGGCTTCTCGTAAGC	-3'
UBP13	CDS	5'- ATGACTATGATGACTCCGCCGCCGCTAGAT	-3'	5'- ATTGTATATTTTCACCGGCTTCTCGTATGC	-3'
BES1	CDS	5'- ATGACGTCTGACGGAGCAA	-3'	5'- ACTATGAGCTTTACCATTTCCAAGCG	-3'
BZR1	CDS	5'- ATGACTTCGGATGGAGCTACG	-3'	5'- ACCACGAGCCTTCCCATTTC	-3'
SINAT2	CDS	5'- ATGGCTCCTGGAGGCAGT	-3'	5'- CTCCTCTTTCCAGATTCGTCCAGT	-3'
UBP12-N	derivatives	5'- ATGACTATGATGACTCCGCCTCCCGTTGAT	-3'	5'- TAAATGTTCTGCTATGTCTTTCTCATC	-3'
UBP12-C	derivatives	5'- CACCAGGGTGAGGCTAAAGAAAGAG	-3'	5'- ATTGTATATTTTTACCGGCTTCTCGTAAGC	-3'
UBP13-N	derivatives	5'- ATGACTATGATGACTCCGCCGCCGCTAGAT	-3'	5'- CAAATGTTCCGCAATGTCTTTC	-3'
UBP13-C	derivatives	5'- CACCCGGGTGAGGCTGAAAAAAG	-3'	5'- ATTGTATATTTTCACCGGCTTCTCGTATGC	-3'
BES1ΔC1	derivatives	5'- ATGACGTCTGACGGAGCAA	-3'	5'- AGCCATGTCACCAGGTAGAG	-3'
BES1ΔC2	derivatives	5'- ATGACGTCTGACGGAGCAA	-3'	5'- ATGGAACTGGCGATGATGAG	-3'
BES1ΔC3	derivatives	5'- ATGACGTCTGACGGAGCAA	-3'	5'- CGAGGTTGGCACCATAGAGG	-3'
BES1ΔN1	derivatives	5'- ATGGGATCATCTTCTCGAGCAACTCC	-3'	5'- ACTATGAGCTTTACCATTTCCAAGCG	-3'
BES1ΔN2	derivatives	5'- ATGGCTCCGGCTACTATACCTGAAT	-3'	5'- ACTATGAGCTTTACCATTTCCAAGCG	-3'
BES1ΔN3	derivatives	5'- ATGCCTACCTTCAATCTCGTGAAACCT	-3'	5'- ACTATGAGCTTTACCATTTCCAAGCG	-3'
BES1_M	derivatives	5'- ATGGGATCATCTTCTCGAGCAA	-3'	5'- ATGGAACTGGCGATGATGAG	-3'
BES1_M1	derivatives	5'- ATGGGATCATCTTCTCGAGCAA	-3'	5'- AGGAAGCGATGAAGGAATACCA	-3'
BES1_M2	derivatives	5'- ATGCCACTTAGAATCTCAAACAG	-3'	5'- ATGGAACTGGCGATGATGAG	-3'
CPD	qRT-PCR	5'- AGACGTGCAATGACGGATGT	-3'	5'- TACCGAGTTGCTCTGCCATC	-3'
DWF4	qRT-PCR	5'- CCCTAGTGGGTGGAAAGTGT	-3'	5'- TCCGTTGTTTTGCTGTTGCC	-3'
SAUR-AC1	qRT-PCR	5'- AGGGAATCATCGTCGACACC	-3'	5'- AAGTATGAAACCGGCACCACA	-3'
ACS5	qRT-PCR	5'- GGACTTCCTGGTTTCCGTGT	-3'	5'- GTCGGAGAGCAATGCAGAGA	-3'
BES1	qRT-PCR	5'- GATGACGTCTGACGGAGCAA	-3'	5'- ACCAGTATAAATCTTCGCCGCA	-3'
UBP12	qRT-PCR	5'- TTGCCAAGTGGAAGTTTGCG	-3'	5'- CAACCCGAGGTACTGCTCAA	-3'
UBP13	qRT-PCR	5'- GGGCAAAGCGTCAAAACCAT	-3'	5'- CGGTCCACGCTCTATTTCCA	-3'
ACT2	qRT-PCR	5'- AGTGGTCGTACAACCGGTATTGT	-3'	5'- GATGGCATGAGGAAGAGAGAAAC	-3'

Table S2 Phenotype changes of various genotypes

Genotypes	Hypocotyl length					Petiole length					Leaf length/width ratio				
	Average	ST	*P*-val	Percent vs WT (%)	Change	Average	ST	*P*-val	Percent vs WT (%)	Change	Average	ST	*P*-val	Percent vs WT (%)	Change
WT	4.9	0.5	1.00	100	0	8.9	0.3	1.00	100	0	2.1	0.1	1.00	100	0
BES1-RNAi	4.1	0.1	0.01	84	-16 ↓	4.8	0.3	0.00	54	-46 ↓	1.2	0.1	0.00	55	-45 ↓
ubp12-2w/13-3	3.2	0.5	0.00	64	-36 ↓	4.8	0.2	0.00	54	-46 ↓	1.4	0.1	0.00	63	-37 ↓
BES1-RNAi x ubp12-2w/13-3	3.3	0.3	0.04	66	-34 ↓	4.6	0.2	0.00	52	-48 ↓	1.3	0.0	0.00	59	-41 ↓
BES1^{OE} *#16*	5.7	0.4	0.01	116	16 ↑	9.9	0.5	0.06	111	11 ↑	2.8	0.1	0.03	131	31 ↑
BES1^{OE} *#18*	6.0	0.5	0.02	123	23 ↑	10.3	0.3	0.01	115	15 ↑	2.9	0.1	0.01	134	34 ↑
UBP12^{OE} *#8*	5.7	0.2	0.12	116	16 ↑	10.5	0.4	0.01	118	18 ↑	2.6	0.2	0.03	123	23 ↑
UBP12^{OE} *#9*	5.5	0.4	0.00	111	11 ↑	10.3	0.2	0.00	115	15 ↑	2.5	0.1	0.03	119	19 ↑
BES1^{OE}*/UBP12*^{OE} *#16*	7.1	0.8	0.00	145	**45** ↑	12.5	0.3	0.00	140	**40** ↑	3.1	0.3	0.01	145	**45** ↑
BES1^{OE}*/UBP12*^{OE} *#23*	7.2	0.6	0.01	146	**46** ↑	12.7	0.5	0.00	143	**43** ↑	3.2	0.2	0.00	151	**51** ↑
BES1^{OE}*/ubp12-2w/13-3 #6*	3.1	0.2	0.19	64	-36 ↓	4.7	0.4	0.00	53	-47 ↓	1.5	0.1	0.05	72	-28 ↓
BES1^{OE}*/ubp12-2w/13-3 #17*	3.5	0.3	0.49	71	-29 ↓	4.9	0.4	0.00	55	-45 ↓	1.6	0.1	0.06	74	-26 ↓
UBP12^{OE}*/RNAi-BES1 #3*	5.0	0.3	0.89	101	1	6.4	0.5	0.00	72	-28 ↓	1.7	0.1	0.01	79	-21 ↓
UBP12^{OE}*/RNAi-BES1 #9*	5.1	0.4	0.45	104	4	6.9	0.4	0.00	77	-23 ↓	1.8	0.2	0.03	84	-16 ↓

↓ Decrease
↑ Increase
